# Iron Oxyhydroxide Transformation in a Flooded Rice Paddy Field and the Effect of Adsorbed Phosphate

**DOI:** 10.1021/acs.est.4c01519

**Published:** 2024-06-04

**Authors:** Katrin Schulz, Worachart Wisawapipat, Kurt Barmettler, Andrew R. C. Grigg, L. Joëlle Kubeneck, Luiza Notini, Laurel K ThomasArrigo, Ruben Kretzschmar

**Affiliations:** † Soil Chemistry Group, Institute of Biogeochemistry and Pollutant Dynamics, CHN, ETH Zurich, Zurich 8092, Switzerland; ‡ Department of Soil Science, Faculty of Agriculture, Kasetsart University, Bangkok 10900, Thailand

**Keywords:** ferrihydrite, lepidocrocite, Mössbauer, iron reduction, microsite, Fe(II)-catalyzed, isotope

## Abstract

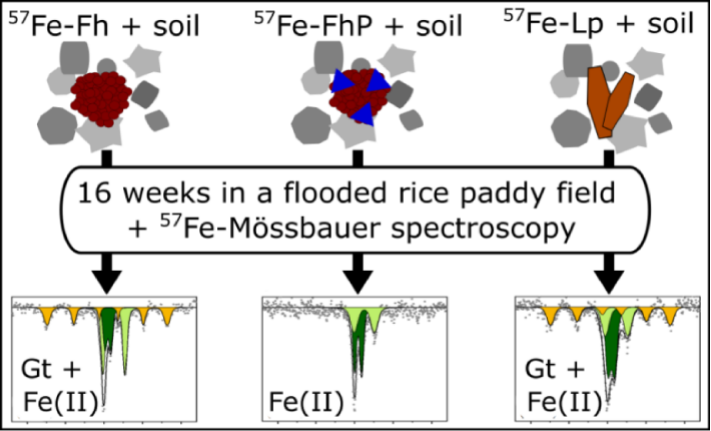

The mobility and bioavailability of phosphate in paddy soils are closely coupled to redox-driven Fe-mineral dynamics. However, the role of phosphate during Fe-mineral dissolution and transformations in soils remains unclear. Here, we investigated the transformations of ferrihydrite and lepidocrocite and the effects of phosphate pre-adsorbed to ferrihydrite during a 16-week field incubation in a flooded sandy rice paddy soil in Thailand. For the deployment of the synthetic Fe-minerals in the soil, the minerals were contained in mesh bags either in pure form or after mixing with soil material. In the latter case, the Fe-minerals were labeled with ^57^Fe to allow the tracing of minerals in the soil matrix with ^57^Fe Mössbauer spectroscopy. Porewater geochemical conditions were monitored, and changes in the Fe-mineral composition were analyzed using ^57^Fe Mössbauer spectroscopy and/or X-ray diffraction analysis. Reductive dissolution of ferrihydrite and lepidocrocite played a minor role in the pure mineral mesh bags, while in the ^57^Fe-mineral–soil mixes more than half of the minerals was dissolved. The pure ferrihydrite was transformed largely to goethite (82–85%), while ferrihydrite mixed with soil only resulted in 32% of all remaining ^57^Fe present as goethite after 16 weeks. In contrast, lepidocrocite was only transformed to 12% goethite when not mixed with soil, but 31% of all remaining ^57^Fe was found in goethite when it was mixed with soil. Adsorbed phosphate strongly hindered ferrihydrite transformation to other minerals, regardless of whether it was mixed with soil. Our results clearly demonstrate the influence of the complex soil matrix on Fe-mineral transformations in soils under field conditions and how phosphate can impact Fe oxyhydroxide dynamics under Fe reducing soil conditions.

## Abstract 2

Adopting a new approach for studying iron mineral transformations in situ in the field, we show that direct soil contact and adsorbed phosphate strongly influence reductive iron mineral transformations.

## Introduction

Phosphorus is an essential plant nutrient which is often yield-limiting,^1^ especially in acidic (sub)tropical soils.^2,3^ Plants take up phosphorus as orthophosphate anions (HPO_4_^2–^, H_2_PO_4_^–^) from the soil solution.^4^ The bioavailability of orthophosphate in soils is often limited due to its association with organic or inorganic soil components.^5^ Iron (Fe) oxyhydroxides are among the most important inorganic sorbents for phosphate in acidic soils and are known to limit phosphate mobility.^6,7^ Since Fe is highly sensitive to changes in soil redox conditions, phosphate mobility and bioavailability can be controlled by Fe oxyhydroxide dynamics in redox-active soils.^8,9^

In rice paddy soils, reducing conditions are frequently established through the flooding of the soil during the rice growing period, which limits the oxygen supply to the soil.^10,11^ Under oxygen limitation, the reduction of alternative electron acceptors, such as ferric iron (Fe(III)), is coupled to the microbial mineralization of organic matter.^12^ By quantity, Fe(III) is one of the most relevant electron acceptors under anoxic conditions in rice paddy soils,^13^ resulting in the reduction of Fe(III) to ferrous iron (Fe(II))^12,14^ and the reductive dissolution of Fe oxyhydroxides, such as ferrihydrite and lepidocrocite.^15^ Microbial Fe reduction can be enhanced by the addition of dissolved phosphate, as shown in mineral slurry experiments.^16,17^ In soils that are limited in available phosphate, the addition of phosphate can stimulate microbial activity and can result in increased Fe(III) reduction rates leading to a faster release of dissolved Fe(II) to the porewater.^18^

The Fe(II) released from microbial Fe reduction can interact with the remaining Fe oxyhydroxides. The Fe(II) adsorbs to Fe oxyhydroxide surfaces and becomes oxidized, transferring an electron to structural Fe(III) which then becomes reduced and is released as Fe(II),^19^ inducing a dissolution–reprecipitation mechanism.^20,21^ For ferrihydrite, a short-range-ordered Fe oxyhydroxide, and lepidocrocite, which is slightly more crystalline, this interaction catalyzes their transformation to more crystalline Fe-minerals, such as goethite^22−24^ or magnetite.^22,24,25^ The trajectory of Fe(II)-catalyzed ferrihydrite and lepidocrocite transformation depends, among other parameters, on the Fe(II):Fe(III) ratio,^22,23,26^ and pH.^22,27^ For example, Fe(II)-catalyzed ferrihydrite and lepidocrocite transformation to magnetite occurs at high Fe(II):Fe(III) ratios and pH ≥ 7.^22,26,28^ At lower Fe(II):Fe(III) ratios, ferrihydrite transforms to lepidocrocite or goethite^22,23^ while lepidocrocite recrystallizes^26^ but remains largely untransformed.^28,29^ In comparison to the catalysis by Fe(II), the microbially mediated transformation of Fe oxyhydroxides leads to the formation of goethite and magnetite^24,25,30^ and the formation of green rusts.^25,30,31^ The microbially driven mineral transformation products depend, among other parameters, on the type and abundance of Fe-reducing bacteria^30,32^ and the Fe(II) production rate and extent.^17,24,31,33^

Although phosphate can enhance microbial Fe reduction,^16−18^ it has been shown that phosphate can hinder microbially mediated Fe-mineral transformations.^16,34^ During microbially mediated ferrihydrite transformation, adsorbed phosphate has been reported to decrease the ferrihydrite transformation extent.^34^ Phosphate occupies ferrihydrite surface sites and favors the formation of green rust and/or vivianite,^16,25,34^ over magnetite^16^ and goethite.^34^ Additionally, phosphate can limit Fe polymerization to Fe(III) oligomers^35^ and thus hinders the formation of crystalline Fe-minerals. The reports that phosphate both increases overall Fe reduction while hindering Fe-mineral transformations suggest that our understanding of the role of phosphate during mineral transformations in soils is incomplete.

Most previous studies have investigated the transformations of Fe oxyhydroxides under controlled laboratory conditions. However, processes under field conditions may be substantially different from those observed in mixed soil or mineral slurries in the laboratory. For example, other soil minerals and organic matter offer additional sorption sites for Fe(II), P, and other solutes, potentially influencing local conditions for Fe-mineral dissolution and precipitation processes. It has recently been demonstrated that direct contact with a soil matrix can strongly influence Fe-mineral transformations.^36^ Additionally, biogeochemical and physical heterogeneities at the pore scale may cause advective flow and diffusion limitations, leading to the development of microsites varying in microbial activity and porewater chemistry. Therefore, we investigated these processes directly in a flooded rice paddy field, exploring (i) ferrihydrite and lepidocrocite transformations and (ii) the role of phosphate during the reductive dissolution and transformation of ferrihydrite. We incubated ferrihydrite, lepidocrocite, and phosphate-adsorbed ferrihydrite in a flooded rice paddy soil in Thailand using mesh bags for 16 weeks. Minerals were additionally incubated as ^57^Fe-labeled mineral–soil mixes. Mineral transformation products were tracked with XRD and/or ^57^Fe Mössbauer spectroscopy, while geochemical conditions in the porewater were monitored.

## Materials and Methods

### Soil Sampling and Characterization

Soil samples were taken during the dry season (February 2020) from a rice paddy field at the Ubon Ratchathani Rice Research Center (URRC) , Thailand. A soil profile with 2 m depth was established in the experiment field site, and the soil was described and classified as a Hydragric Loamic Anthrosol after the World Reference Base for Soil Resources.^37^ The soil showed typical^10^ rice paddy features, such as a puddled horizon, including a dense plow pan and distinct hydromorphic features in the subsoil. A description of the soil profile is presented in the Supporting Information, Section S1. Approximately 10 kg of topsoil (0–15 cm) was taken for preparing the mineral–soil mixes, and small soil samples in 10 cm increments were taken for soil characterization. The soil samples were oven-dried at 30 °C until constant weight, before all samples were homogenized by sieving (<2 mm), and aliquots were milled with a vibratory disc mill. The texture of the sieved topsoil (0–15 cm) was silty sand (2.6% clay, 12.6% silt, 84.8% sand). The total element contents in the topsoil were determined in a previous study to be 3.3 g Fe kg soil^–1^ and 4.0 g C kg soil^–1^.^36^ Depth-resolved element contents are presented in Figure S2. Total phosphorus (0.08 g kg^–1^)^36^ contents were measured after the total digestion (hydrofluoric acid) of the soil.^38^ The pH of the topsoil (0–15 cm) in 0.01 M CaCl_2_ was weakly acidic (pH 5.5). The Fe mineralogy in the topsoil (0–15 cm) has been characterized previously using Mössbauer spectroscopy and a five-step sequential extraction.^36^ These analyses showed that ferrihydrite and goethite were the main Fe-mineral phases in the soil, along with silicate mineral/organic matter-associated Fe(III) and silicate mineral-associated/adsorbed Fe(II).^36^ Mössbauer spectra of the soil are presented in Figure S3.

### Mineral Synthesis and Characterization

Ferrihydrite and lepidocrocite with natural abundance (NA) Fe isotope composition (5.9% ^54^Fe, 91.7% ^56^Fe, 2.1% ^57^Fe, 0.3% ^58^Fe)^39^ were synthesized following the methods of Schwertmann and Cornell.^40^ Isotopically labeled ferrihydrite (^57^Fe-Fh) and lepidocrocite (^57^Fe-Lp) were synthesized with slightly modified methods using ^57^Fe(0) (96.14% ^57^Fe, Isoflex USA) dissolved in 2 M HCl (NORMATOM, 34–37%, VWR) and oxidized with H_2_O_2_ (35%, Merck). A detailed description of the synthesis of ferrihydrites and lepidocrocites is presented in Section S2. To obtain phosphate-adsorbed ferrihydrite (^NA^Fe-FhP and ^57^Fe-FhP), the ^NA^Fe-Fh and ^57^Fe-Fh were resuspended in ultrapure water (UPW, >18.2 MΩ·cm, Milli-Q, Merck Millipore). The suspensions were spiked with a phosphate solution, derived from Na_2_HPO_4_ (VWR) at a molar ratio of P/Fe = 0.1 (1.2 mmol P per g ferrihydrite). The pH was adjusted to pH 6.5 ± 0.1 using 1 M NaOH. The suspensions were mounted on an overhead shaker for 40 h. Since the pH slightly increased during the adsorption (pH up to approximately 6.7–6.8), the pH was readjusted to pH 6.5 ± 0.1 after 12, 24, and 36 h using 1 M HCl. The ^NA^Fe-FhP and ^57^Fe-FhP suspensions were washed, centrifuged, dried, and homogenized as described for ^NA^Fe-Fh and ^57^Fe-Fh (Section S2). The P concentration measured by inductively coupled plasma optical emission spectrometry (ICP-OES, Agilent 5100) in the supernatant of the ferrihydrite-P suspensions after 40 h was below detection limit. The final molar P/Fe ratio in the ^NA^Fe-FhP and ^57^Fe-FhP solid phases was 0.1, as determined with ICP-OES after mineral dissolution in concentrated HCl at room temperature. All minerals were characterized by X-ray diffraction (XRD), which confirmed the expected mineral composition and found no evidence of crystalline impurities (Figure S4). For the lepidocrocites, Mössbauer spectroscopy (5 K) indicated that ^NA^Fe-Lp and ^57^Fe-Lp contained small fractions (7% in ^NA^Fe-Lp, 16% in ^57^Fe-Lp) of goethite (Figure S5).

### Sample Preparation and Sample Holders

Minerals and mineral–soil mixes were incubated in the soil using mesh bags which were prepared from a polyethylene terephthalate (PETE) filter fabric (internal dimensions ∼1 × 3 × 0.3 cm, pore size 51 μm; SEFAR, Switzerland). The mesh bags were made by folding the triple-layered filter fabric and heat sealing it on two sides, before the minerals or mineral–soil mixes were filled into the bag. For the ^NA^Fe-minerals, 100 mg of the dried mineral powders (^NA^Fe-Fh, ^NA^Fe-FhP, or ^NA^Fe-Lp) was weighed and filled into each mesh bag before the mesh bag was closed by heat sealing. For the ^57^Fe-mineral–soil mixes, 10 mg of ^57^Fe-Fh, ^57^Fe-FhP, or ^57^Fe-Lp was mixed with 800 mg of the dried and sieved topsoil and filled into the mesh bags as described above. The chosen amount of ^57^Fe-mineral and soil in the ^57^Fe-mineral–soil mixes aimed at minimizing the addition of Fe to the soil (mineral addition increased the soil Fe content 3.7 times) while obtaining an adequately high Mössbauer signal from the ^57^Fe-minerals.^41^ In this study, >98% of the total ^57^Fe in the initial mineral–soil mixes came from the added ^57^Fe-labeled ferrihydrite or lepidocrocite, ensuring that the spiked mineral dominated the Mössbauer signal. The mesh bags were mounted into 3D-printed (photopolymer resin, Formlabs) sample holders, and a threaded plastic rod was attached. The sample holders enabled the insertion of the samples into the soil at a defined depth and allowed the contact between the mesh bag and the surrounding soil through large vertical openings on the side (picture in Figure S7).

### Experimental Setup and Sampling

The 16-week-long field incubation of Fe-minerals was performed during the wet season in July 2022 in the same paddy field which was sampled for soil characterization in 2020 (URRC, Thailand). At the start of the experiment, the soil had been in a flooded state for 2 weeks, and the soil was water saturated, with approximately 1 cm of water above the soil surface. Prior to starting the experiment and at each sampling (8, 12, and 16 weeks), rice plants and weeds were manually removed from the experiment site (2.5 × 6.5 m). Additionally, the soil surface was manually leveled prior to starting the experiment. The experiment was set up in triplicate with three circular-shaped plots within the experiment site, each containing an equal set of samples (experimental scheme in Figure S6). The sample holders containing the mesh bags were equally distributed among the plots and installed in the soil at 15 cm depth. Three soil porewater samplers (MacroRhizons, pore size 0.15 μm, Rhizosphere) were installed at 15 cm below the soil surface for the duration of the experiment.

Porewater (∼25 mL) was extracted using the installed porewater samplers at the start of the experiment and after 8, 12, and 16 weeks. The pH was measured with a glass electrode (Metrohm) in ∼10 mL porewater immediately after porewater extraction. Aliquots of porewater samples were immediately stabilized by either adding concentrated HCl for total element concentration analysis with ICP-OES or by adjusting the pH to ∼3–4 with 1 M HCl for dissolved organic carbon analysis (DIMATOC 2000, Dimatec). Depth-resolved oxidation–reduction potential (ORP) measurements were taken in duplicate using custom-made Eh probes (Paleoterra, Pt-electrodes, Ag/AgCl saturated KCl reference electrode) after equilibration in the soil for ≥8 h. The ORP readings were converted to redox potentials relative to the standard hydrogen electrode (Eh) by the addition of +189 mV (saturated KCl, 32 °C). The temperature was measured manually in the soil at sample depth (15 cm). At every sampling (8, 12, and 16 weeks), one set of mesh bags (^NA^Fe-Fh, ^NA^Fe-FhP, ^NA^Fe-Lp, ^57^Fe-Fh, ^57^Fe-FhP, ^57^Fe-Lp) was removed from each replicate plot. The samples were vacuum sealed immediately in the field to avoid oxidation of Fe(II). Subsequently, all samples were additionally sealed in Al bags under nitrogen flow in the field. Samples were stored frozen (−18 °C), shipped to Zurich (Switzerland), and air-dried under glovebox N_2_ atmosphere (MBraun).

### Solid Phase Analyses

The transformation of ferrihydrite and lepidocrocite in ^NA^Fe-mineral samples without soil and in the ^57^Fe-mineral–soil mixes was tracked by Mössbauer spectroscopy, which is only sensitive to ^57^Fe, and therefore, selectively reveals the speciation of ^57^Fe in the sample. For Mössbauer analysis, the triplicate samples were combined, and spectra were collected at 77 and 5 K. Further details on Mössbauer sample preparation and measurements are presented in Section S6.

The ^NA^Fe-mineral samples without soil were additionally analyzed by X-ray diffraction (XRD, Bruker D8 Advance), and mineral phase contributions were quantified using Rietveld quantitative phase analysis of diffraction patterns. Ferrihydrite was included as a mass-calibrated PONKCS^42^ phase in the fits. Further details on sample preparation, measurements, and the fitting of the XRD patterns are presented in Section S7.

To determine the elemental composition of initial and incubated ^NA^Fe-mineral samples, triplicate samples were combined and dissolved in concentrated HCl (NORMATOM, 34–37%, VWR) at room temperature before the solutions were passed through a nylon filter (<0.45 μm). The filtrates were analyzed for total element contents by ICP-OES. For ^57^Fe-mineral–soil mixes, potential changes in the Fe content and the Fe isotope fractions were analyzed following aqua regia digestion. For the digestion, representative aliquots (∼150 mg) of homogenized samples were weighed into 15-mL centrifuge tubes. Freshly prepared aqua regia (10 mL, HNO_3_:HCl ratio 1:3) was added to each vial, and the digestion was conducted at 120 °C for 90 min. Digested samples were passed through a 0.45 μm PTFE filter, and total Fe concentrations in the filtrates were measured by ICP-OES. To analyze the Fe isotope composition, the filtrates were diluted to 50 ppb Fe and analyzed with triple-quadrupole inductively coupled plasma mass spectrometry (ICP-MS, Agilent 8800 Triple Quad). The ^57^Fe isotope fractions were calculated relative to the sum (counts per second) of ^54^Fe, ^56^Fe, ^57^Fe, and ^58^Fe.^26,43^

## Results and Discussion

### Soil and Porewater Conditions

**Figure 1:**
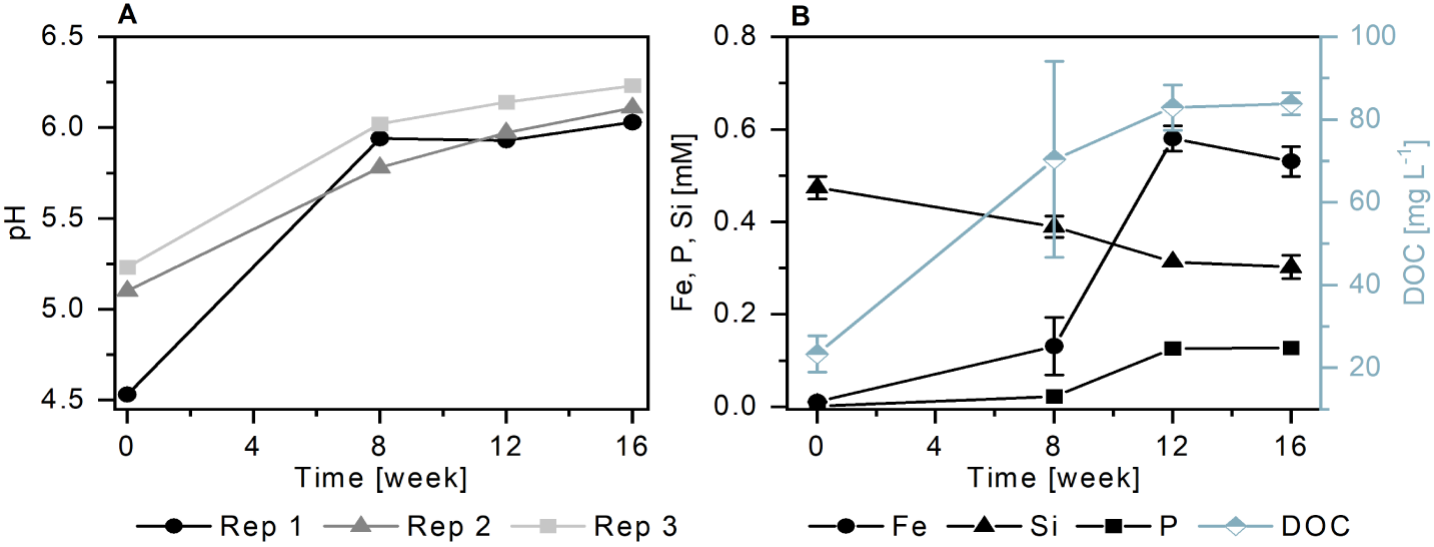
pH of the porewater at the start of the experiment (week 0) and the sampling points at 8, 12, and 16 weeks (A), and porewater concentrations of iron (Fe), phosphorus (P), silicon (Si), and dissolved organic carbon (DOC) (B). Error bars in panel B show the standard error between triplicate porewater samples, errors <0.015 mM for Fe, P, and Si concentrations or <3 mg L^–1^ for DOC concentrations are smaller than symbols and are not shown. Porewater concentrations of dissolved magnesium, sodium, sulfur, and calcium are presented in Figure S9. Abbreviation: Rep = replicate.

At the start of the experiment, the Eh and pH at the sample depth (15 cm) in the flooded soil ranged between +1 and −105 mV (*n* = 2, Figure S8) and pH 4.5 to 5.2 (*n* = 3, Figure 1A). In the following weeks, the spatial variability in Eh and pH conditions diminished. Throughout 16 weeks, the Eh dropped further and ranged between −113 and −152 mV, while the pH increased and ranged between pH 6.0 and 6.2. The Eh at sample depth was well within the range where Fe reduction is possible (below approximately +100 mV at pH 7),^10^ and the soil temperature measured at sample depth (15 cm) was stable at 31.7 ± 2.7 °C (mean 0, 8, 12, 16 weeks ± standard error of the mean) for the duration of the experiment.

Dissolved Fe was released to the porewater in the flooded soil during the 16-week experiment (Figure 1B). While no dissolved Fe was detected in the porewater at the start of the experiment (0 weeks), the Fe concentration was low at 8 weeks (0.1 mM), reached a maximum at 12 weeks (0.6 mM), and then remained in a similar range until the end of the experiment (16 weeks, 0.5 mM). Similar to Fe, dissolved P was not detected in the porewater at the start of the experiment (0 weeks), the concentration was low at 8 weeks (<0.03 mM) and increased up to 0.1 mM at 16 weeks (Figure 1B). Since the vast majority of dissolved Fe in flooded soil porewaters is present as Fe(II),^41,44^ the similar release patterns of Fe and P suggest that Fe(III) phases in the soil were reductively dissolved, releasing Fe(II) and mineral-associated oxyanions,^45,46^ including phosphate.^8,9^ Alternatively, P may have been released through the decomposition of organic matter and/or desorption from the soil.

Other elements were already present in the porewater at the start of the experiment (0 weeks), such as C (23.3 mg L^–1^ DOC), Si (0.5 mM), Mg (0.2 mM), Na (3.5 mM), S (0.7 mM), and Ca (0.5 mM) (Figures 1B and S9). The concentration of DOC increased throughout the experiment (84 mg L^–1^ at 16 weeks, Figure 1B), likely due to release from reductively dissolved Fe-minerals,^47^ desorption, and the decomposition of organic matter. The concentrations of Si, Mg, Na, Ca, and S decreased after the start of the experiment. Dissolved S concentrations were below detection limit after the initial time point (0 weeks). No dissolved Mn was detected in the porewater.

### Iron Phases Identified with Mössbauer Spectroscopy

**Figure 2:**
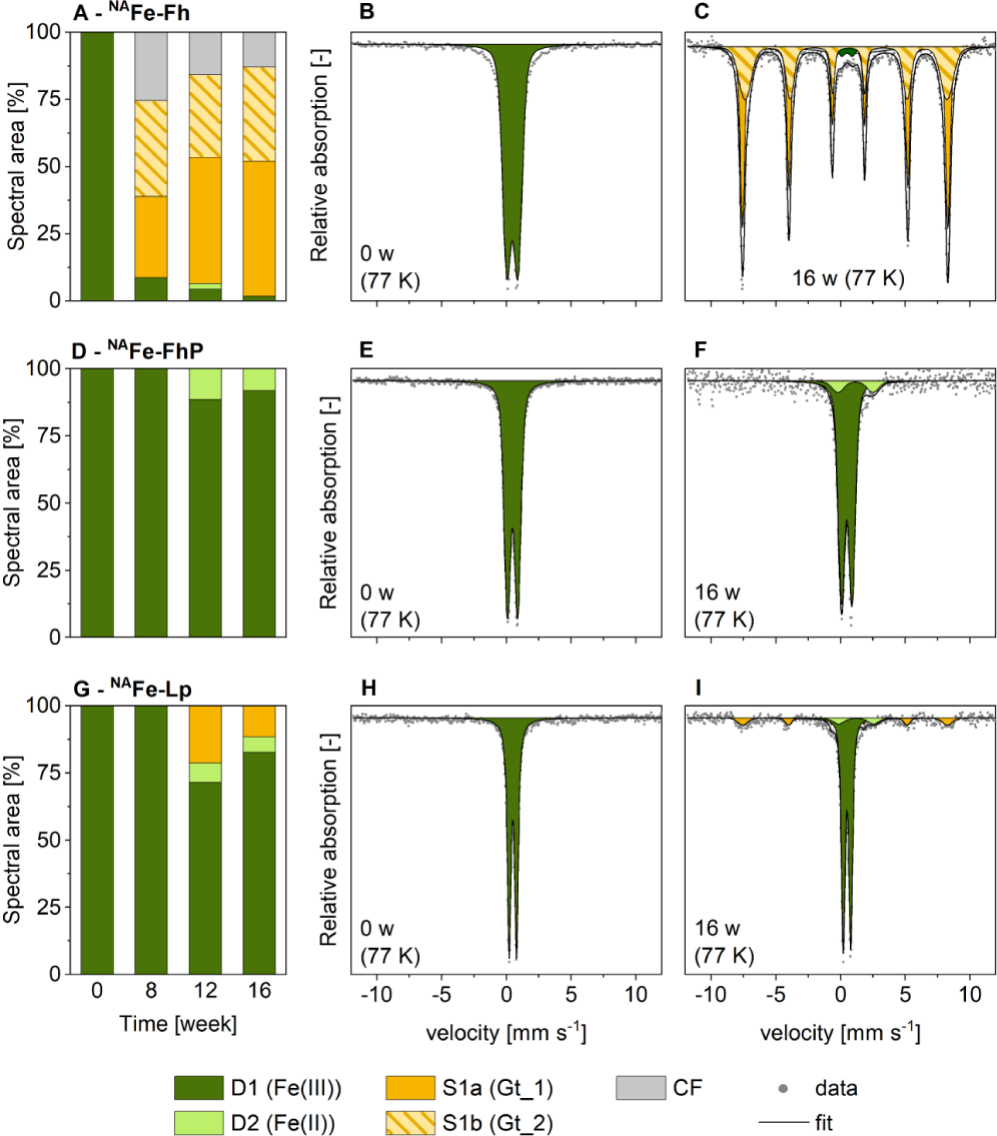
Fitted Fe phase fractions (A,D,G) and corresponding Mössbauer spectra collected at 77 K of initial (B,E,H) and 16-week incubated (C,F,I) ^NA^Fe-mineral samples without soil for the ^NA^Fe-Fh (A-C), ^NA^Fe-FhP (D–F) and ^NA^Fe-Lp (G–I)–soil mixes. All fit components are presented in Table 1. Abbreviations: w = weeks, Gt = goethite, CF = collapsed feature. Fitting parameters and spectra from other time points are presented in Section S6.

**Figure 3:**
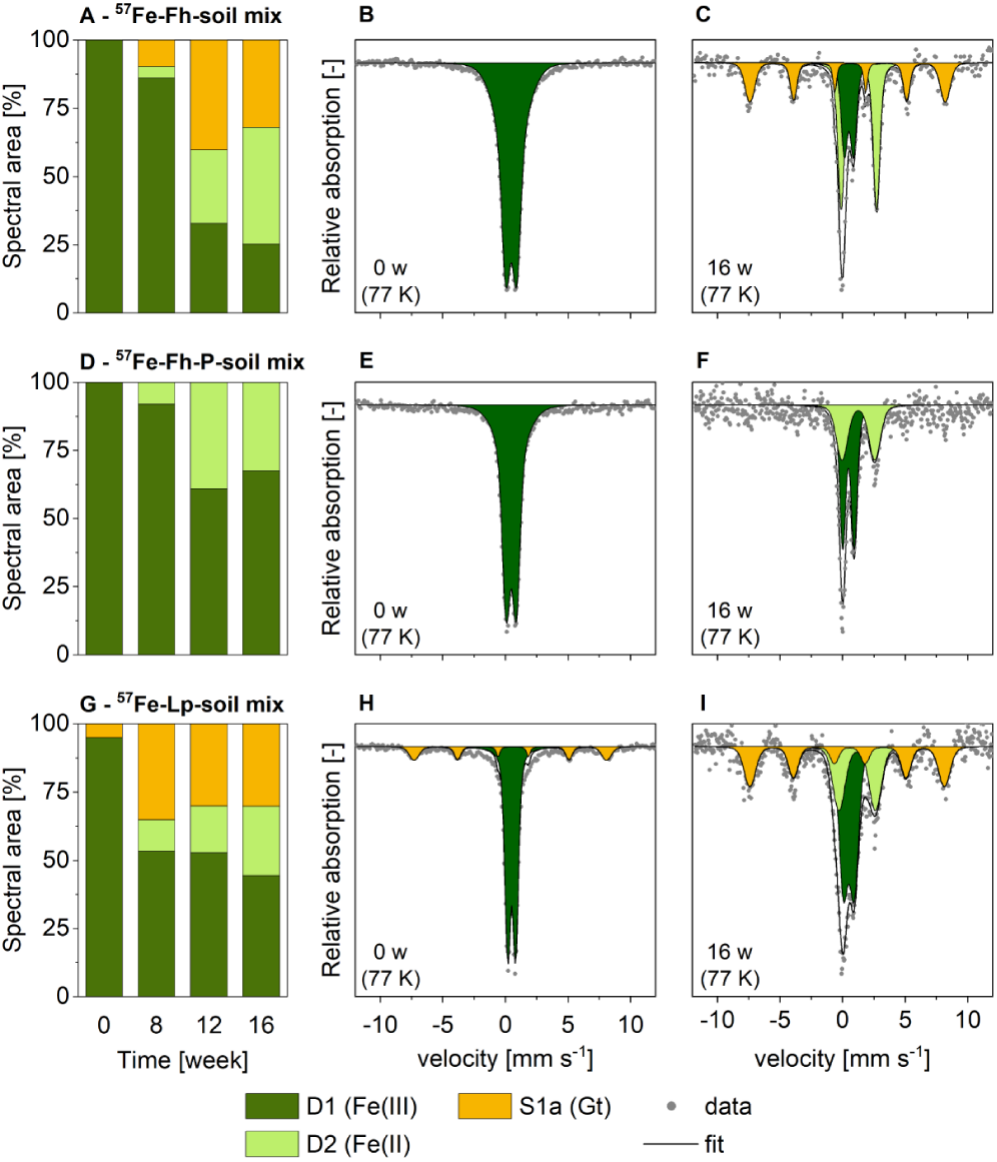
Fe phase fractions (A,D,G) and corresponding Mössbauer spectra collected at 77 K of initial (B,E,H) and 16-week incubated (C,F,I) ^57^Fe-mineral–soil mixes, for the ^57^Fe-ferrihydrite (A–C), ^57^Fe- ferrihydrite-P (D–F) and ^57^Fe-lepidocrocite (G–I)–soil mixes. All fit components are presented in Table 1. Abbreviations: w = weeks, Gt = goethite. Interpretation of fit components are summarized in Table 1. Fitting parameters and spectra from other time points are presented in Section S6.

The Fe speciation in initial and incubated ^NA^Fe-mineral samples and ^57^Fe-mineral–soil mixes was analyzed with Mössbauer spectroscopy. The 77 K spectra were fit with the components presented in Table 1, where averaged values for center shift (CS), quadrupole splitting (QS, for doublets), or quadrupole shift (ε, for sextets) and hyperfine field (H) are reported. Fit parameters for all samples are presented in Section S6. Mössbauer spectra collected at 77 K from initial ferrihydrite and lepidocrocite in the ^NA^Fe-mineral samples without soil (Figure 2BH) and the ^57^Fe-mineral–soil mixes (Figure 3BH) showed a doublet (D1) with fitting parameters in agreement with those of paramagnetic Fe(III).^48^ At 77 K, paramagnetic Fe(III) includes Fe in ferrihydrite^49^ and lepidocrocite^50^ but also organic matter-complexed or silicate-associated Fe(III).^48^

**Table 1:** Fit Components (D = Doublet, S = Sextet, CF = Collapsed Feature) for the Fitting of Mössbauer Spectra Collected at 77 K from ^NA^Fe-Minerals and ^57^Fe-Mineral–Soil Mixes, with Averaged Fitting Parameters,^a^ Corresponding Interpretations and References

fit components	CS^b^ [mm s^–1^]	QS^c^ or ε^d^ [mm s^–1^]	H^e^ [T]	interpretation	references
doublet D1	0.48	0.71	-	paramagnetic Fe(III), e.g., in ferrihydrite and lepidocrocite, or complexed/silicate-associated Fe(III)	(48−50)
doublet D2	1.20	2.70	-	solid-associated Fe(II), e.g., in primary minerals, silicate-associated or adsorbed	(65)
sextet S1a	0.49	–0.12	49.20	Fe(III) in goethite (Gt_1)	(52)
sextet S1b	0.46	–0.09	48.70	Fe(III) in goethite with lower crystallinity (Gt_2)	(48)
collapsed feature (CF)	0.80	0.00	46.67	Fe in Fe phases near their blocking temperature	(65)

a All fitting parameters are presented in Section S6.

b Center shift.

c Quadrupole splitting (for doublets).

d Quadrupole shift (for sextets).

e Hyperfine field.

In addition to the paramagnetic Fe(III) doublet D1, the fits of 77 K Mössbauer spectra from incubated ^NA^Fe-Fh samples without soil required the inclusion of a collapsed feature (Figure S10). This collapsed feature indicates the presence of an Fe phase with a blocking temperature around 77 K. Mössbauer spectra collected at 5 K, suggested that ferrihydrite, lepidocrocite, and goethite were the only Fe-mineral phases in the incubated ^NA^Fe-mineral samples (Figure S11). This observation is in agreement with XRD results (Figure S14A,B). Therefore, the collapsed feature in 77 K spectra of ^NA^Fe-Fh was interpreted as an Fe oxyhydroxide phase with a low crystallinity, such as ferrihydrite or nanogoethite.

In incubated ^57^Fe-mineral–soil mixes, a prominent doublet with fitting parameters suggesting paramagnetic Fe(II) (D2) appeared in 77 K Mössbauer spectra. At 5 K, small fractions of a collapsed feature were present in the spectra (Figure S13). A similar collapsed feature in 5 K Mössbauer spectra has been interpreted as a highly disordered Fe phase in previous studies^48,51^ and in a recent laboratory incubation study using a similar soil from the same experimental field.^36^

### Mineral Transformations: Pure ^NA^Fe-Minerals

The pure ferrihydrite in mesh bags without soil (^NA^Fe-Fh) showed a high extent of transformation to more crystalline Fe-minerals, as evidenced by Mössbauer results (77 K data in Figures 2 and S10; 5 K data in Figure S11) and XRD (Figure S14). According to Mössbauer spectroscopy, 85% of ^NA^Fe-Fh transformed to goethite within 16 weeks (Figure 2C). Mössbauer spectra (77 K) of ^NA^Fe-Fh showed two sextets (S1a and S1b, with parameters similar to those reported for goethite).^48,52^ Sextet S1b had a less negative quadrupole shift (−0.09 mm s^–1^) compared to sextet S1a (−0.13 mm s^–1^), which suggests that sextet S1b represents a phase with lower crystallinity, such as nanogoethite.^48^ In good agreement with Mössbauer results, XRD and Rietveld analysis indicated 82% goethite in this sample (Figure S14A,B). We suggest that the extent of microbial Fe reduction inside the mesh bags was small relative to total Fe in the mesh bags. Even though Fe-reducing bacteria may have passed the mesh, there was no organic substrate inside the mesh bags initially that could have been oxidized. Any DOC that diffused into the mesh bags during the incubation was likely trapped by the minerals at the rim of the mesh bags.^44^ The limited microbial Fe reduction in ^NA^Fe-mineral samples was supported by minor fractions (≤2%) of solid-associated Fe(II) (doublet D2) in Mössbauer spectra collected from 16-week samples (Figure 2). Therefore, we suggest that the ^NA^Fe-Fh transformation to goethite was likely mainly driven by Fe(II) that diffused into the mesh bags from the surrounding soil and initiated electron transfer.^44^ This is in agreement with observations in abiotic mineral transformation studies involving Fe(II) interactions with minerals in slurries.^23,26,53^

Compared to the short-range-ordered ^NA^Fe-Fh, the more crystalline ^NA^Fe-Lp showed less transformation (compare Figure 2A with Figure 2G). The ^NA^Fe-Lp transformed to 12% goethite (at 16 weeks), as determined by Mössbauer spectroscopy (Figure 2G,I), but no goethite was detected by XRD (Figure S14 E,F). Therefore, the majority of goethite that formed from ^NA^Fe-Lp most likely was goethite with low crystallinity, which contrasts crystalline goethite formation in ^NA^Fe-Fh (Figure S14 A,B). The much smaller extent of mineral transformation in ^NA^Fe-Lp (12% goethite), compared to ^NA^Fe-Fh (>80% goethite) reflects the higher stability of lepidocrocite against Fe(II)-catalyzed mineral transformation,^26,28,29^ compared to ferrihydrite. Despite the limited mineral transformation, the remaining lepidocrocite in ^NA^Fe-Lp may have recrystallized.^26^ Generally, our results of lepidocrocite transformation to goethite agree with observations from mineral slurry experiments.^22,54^ However, the transformation of lepidocrocite in this field study was much slower compared to mineral slurry experiments, where lepidocrocite transformation occurs within hours.^29^ The slower transformation of lepidocrocite may be related to diffusion limitations and thus the lower availability of Fe(II) in the field, compared to agitated slurry experiments. Recently, a study including incubations of mineral-filled mesh bags in soil mesocosms, using a rice paddy soil from the same field as used in this study, reported magnetite formation from ferrihydrite and lepidocrocite.^36^ However, no magnetite was observed in this field experiment. This may be due to less favorable conditions for magnetite formation, including lower dissolved Fe(II) concentrations^22,24^ or lower pH^22^ (pH 6.9 in ref (36) compared to pH 6.2 in this experiment).

Since ^NA^Fe-minerals in this study were exposed to natural soil porewaters (Figure 1), the potential impact of other dissolved soil components on mineral transformations was considered. Element contents measured in dissolved mineral phases indicated that P and Si adsorbed to ^NA^Fe-Fh and ^NA^Fe-Lp during the incubation (Figure S15). The P and Si contents in the mineral phases were similar in ^NA^Fe-Fh and ^NA^Fe-Lp (<0.1 μmol mg^–1^) and corresponded to molar P/Fe and Si/Fe ratios of ∼0.01 at 16 weeks. These ratios are low compared to other studies using synthesized P- or Si-ferrihydrites (e.g., mol ratios of P/Fe = 0.02–0.1, ref (55); Si/Fe = 0.02–0.4, refs (26, 55), and (56); and the phosphate-adsorbed ferrihydrite used in this study, P/Fe = 0.1). Concentrations of other elements present in the porewater, such as Al, Na, Mg, and S were below detection limit in the ^NA^Fe-mineral samples. Additionally, the interactions of the ^NA^Fe-minerals with dissolved porewater components were likely limited to the mineral–soil interface at the rim of the mesh bags.^44^ By mapping cross sections of incubated pure mineral mesh bags with Raman spectroscopy, Grigg et al.^44^ demonstrated that various porewater components adsorbed to the outermost mineral layer, likely due to the large sorption capacity of the synthesized ferrihydrite. Thus, we conclude that dissolved porewater components, other than Fe(II), likely only marginally affected the extent and products of mineral transformations in the ^NA^Fe-mineral mesh bags without soil.

### Mineral Transformations: ^57^Fe-Minerals Mixed with Soil

In contrast to ^NA^Fe-mineral samples without soil, in the ^57^Fe-mineral–soil mixes, the added Fe-minerals only comprised a small fraction of the sample (10 mg mineral in 800 mg soil). Therefore, we combined the use of ^57^Fe-labeled minerals and ^57^Fe Mössbauer spectroscopy to track Fe-mineral transformations in the mineral–soil mixes.^41^ The results showed increasing fractions of solid-associated Fe(II) (D2; Figures 3 and S12), indicating that all ^57^Fe-minerals were partly reductively dissolved during the field incubation of the ^57^Fe-mineral–soil mixes. At 16 weeks, the solid-associated Fe(II) fraction was 43% in ^57^Fe-Fh and 26% in ^57^Fe-Lp and thus was much larger compared to the ^NA^Fe-mineral mesh bags without soil (0% in ^NA^Fe-Fh, 6% in ^NA^Fe-Lp). Even at 5 K, the Fe(II) fraction in the Mössbauer spectra remained present as a doublet, suggesting that this fraction mainly comprised silicate-associated or adsorbed Fe(II).^57^ The reductive dissolution of ^57^Fe-minerals was likely followed by a diffusion of ^57^Fe(II) out of the mesh bags, as it was also observed by Schulz et al.^36^ This was indicated by decreasing Fe contents and decreasing ^57^Fe fractions, as determined in aqua regia-digested ^57^Fe-mineral–soil mix samples (Figure S16). More than half of the ^57^Fe from the ^57^Fe-mineral–soil mixes was lost, leading to increased spectral noise and increased contributions of native soil ^57^Fe to the Mössbauer signal. In turn, contributions of ^57^Fe from the added ^57^Fe-labeled minerals to the Mössbauer signal decreased. At the end of the experiment (16 weeks), the estimated contribution of the remaining ^57^Fe from the added minerals was 74 ± 12% in ^57^Fe-Fh, 76 ± 7% in ^57^Fe-Lp, and 48 ± 19% in ^57^Fe-FhP (assuming that no soil–^57^Fe was lost; Figure S17). The Mössbauer spectra of the dried initial soil (without added ^57^Fe-minerals) collected at 77 K showed an Fe(III) doublet (D1, 54%), a goethite sextet (S1a, 34%), and small fractions of solid-associated Fe(II) (D2, 11%, Figure S3). Given the increasing contribution of soil–^57^Fe to Mössbauer spectra collected from incubated ^57^Fe-mineral–soil mixes, a contribution of these components needs to be considered. Therefore, reported fractions of goethite, Fe(II), and Fe(III) may be slightly overestimated. In fits of Mössbauer spectra collected at 5 K, individual parameters had to be fixed due to high spectral noise and overlapping sextets.

Goethite was formed in ^57^Fe-Fh and ^57^Fe-Lp mineral–soil mixes, as indicated by the sextet (S1a) in 77 K Mössbauer spectra (Figures 3 and S12). Goethite formation from ^57^Fe-Lp and ^57^Fe-Fh occurred already within the first 8 weeks of the incubation (Figure 3A,G), when dissolved Fe concentrations in the soil porewater were still low (Figure 1B, 0.1 mM Fe). After 16 weeks, around one-third of the remaining ^57^Fe atoms were present as goethite in ^57^Fe-Fh and ^57^Fe-Lp-mineral–soil mixes. Accounting for the ^57^Fe that was lost during the incubation (∼65%, Figure S16), this corresponds to ∼10% of the initial ^57^Fe being present as goethite in the ^57^Fe-Fh and ^57^Fe-Lp-mineral–soil mixes after 16 weeks. Since the overall trends are similar, from here on, reported fractions of fit components are discussed in terms of fractions of the remaining ^57^Fe in the solid phase. Hence, we defined the “transformed” mineral fraction in this study as the fraction that remained in the mesh bag and transformed to other Fe-minerals. The solid-associated Fe(II) and the Fe(II) that diffused out of the mesh bag were considered as the “reductively dissolved” Fe fraction.

The presence of ^57^Fe as goethite in the ^57^Fe-mineral–soil mixes may partly be explained by the Fe atom exchange of dissolved ^57^Fe(II), derived from reductively dissolved ^57^Fe-minerals, with goethite in the soil (Figure S3).^58^ However, this mechanism alone cannot account for the extent of goethite formation in this experiment, as also discussed by Notini et al.^41^ Therefore, the transformation of ^57^Fe-labeled ferrihydrite and lepidocrocite to goethite was likely catalyzed by the Fe(II) derived from microbial Fe reduction inside the mesh bags, including reduction of the spiked mineral and native minerals. This is in agreement with results from mineral slurry experiments^22,29,54^ and incubations of ferrihydrite-filled mesh bags in soil.^44,59^

The transformation of ^57^Fe-Fh to goethite in this experiment contrasts the results from recent incubations of ^57^Fe-ferrihydrite–soil mixes. For example, Notini et al.^41^ incubated water-saturated ^57^Fe-ferrihydrite–soil mixes in centrifuge tubes for 12 weeks using a similar rice paddy soil compared to this study and found the formation of a green-rust(-like) phase. In laboratory soil mesocosms, ^57^Fe-ferrihydrite– and lepidocrocite–soil mixes were incubated for up to 12 weeks using mesh bags of similar dimensions and soil from the same paddy field where this field study was conducted.^36^ The study using soil mesocosms found that a mixed-valence highly disordered Fe phase formed in ferrihydrite–soil mixes,^36^ as suggested by a collapsed feature in 5 K Mössbauer spectra.^48,51^ Goethite only formed in lepidocrocite–soil mixes.^36^ Also in the current field study, the fit of Mössbauer spectra collected at 5 K required the inclusion of a collapsed feature. Fractions of the collapsed feature at 16 weeks were much smaller (<20% of ^57^Fe) in this experiment compared to observations in the soil mesocosm incubation (up to 48% of ^57^Fe).^36^ That the trajectory of ferrihydrite transformation in soil differed between this study (^57^Fe-Fh–soil mixes) compared to previous studies^36,41^ may be related to differences in geochemical conditions. These may comprise porewater element concentrations, including the Fe(II) concentration,^22,23,28^ and the rate of microbial Fe reduction.^17,24,41^ For example, the lack of advective flow in soil mesocosms may cause a local accumulation of Fe(II) at anoxic microsites.^36^ Combined with the close association with other dissolved porewater components, this may have promoted the formation of mixed-valent disordered Fe phases.^36^ Additionally, the pH in the anoxic soils was weakly alkaline (up to pH 7.9)^41^ or circumneutral (pH 6.9)^36^ in previous experiments but weakly acidic in this field experiment (pH 6.2, Figure 1A). The lower pH in this experiment may have promoted goethite formation.^22,27^

### The Effects of Adsorbed Phosphate

The adsorbed phosphate in ^NA^Fe-FhP mesh bags strongly hindered ferrihydrite transformations. This was indicated by the absence of crystalline Fe products from ferrihydrite transformation at 16 weeks, as shown by both Mössbauer spectroscopy (Figure 2D,F) and XRD (Figure S14C,D). This agrees with recent results from Kraal et al.^60^ who observed negligible transformation of ferrihydrite-phosphate coprecipitates in phosphate-rich Fe-reducing sediments after 4 weeks. The absent transformation of ^NA^Fe-FhP in this experiment may be explained by adsorbed phosphate occupying the ferrihydrite surfaces^55,60,61^ and thus hindering the interactions with dissolved Fe(II). Additionally, phosphate likely hindered the formation of more crystalline Fe oxyhydroxides by limiting the Fe polymerization to Fe(III)-oligomers.^35^

Similar to ^NA^Fe-mineral mesh bags without soil, adsorbed phosphate in the ^57^Fe-FhP–soil mixes strongly hindered ferrihydrite transformation to goethite, as indicated by 77 K Mössbauer spectra (Figure 3). We did not observe vivianite formation; however, we cannot exclude the formation of minor amounts of vivianite, which may be hidden within the slightly broadened Fe(II) doublet (D2; Figure 3F). In addition to the hindered mineral transformation, the loss of ^57^Fe from the ^57^Fe-FhP–soil mixes with adsorbed phosphate tended to be slightly higher compared to ^57^Fe-Fh–soil mixes (Figure S16). This suggests that adsorbed phosphate may have enhanced the reductive dissolution of ferrihydrite. Increased Fe reduction rates have been reported from soil suspension experiments with the amendment of phosphate.^18^ Similarly, the addition of dissolved phosphate to mineral slurries has been reported to promote the microbially mediated reduction of ferrihydrite.^16^ In conclusion, despite the potential for adsorbed phosphate to promote microbial Fe reduction, the hindrance of ferrihydrite transformation by adsorbed phosphate in the ^57^Fe-FhP–soil mixes persisted. This suggests a dual role of phosphate during in situ ferrihydrite transformations in soil.

The exposure of phosphate-adsorbed ferrihydrite to the Fe reducing porewater in the rice paddy field did not lead to a net loss of P from ^NA^Fe-FhP mesh bags without soil (Figure S15). The molar P/Fe ratio in the ^NA^Fe-FhP mesh bags without soil was 0.11 at 16 weeks. This suggests that phosphate remained associated with ferrihydrite. In comparison to ^NA^Fe-FhP mesh bags without soil, the release of phosphate from ^57^Fe-FhP–soil mixes may have been impacted by in situ reductive dissolution of ferrihydrite. However, since mineral transformation of phosphate-adsorbed ferrihydrite in ^57^Fe-FhP–soil mixes was strongly hindered throughout the 16 weeks, it is likely that phosphate remained associated with ferrihydrite. This is supported by observations from the microbially mediated transformation of phosphate-adsorbed ferrihydrite in minerals slurries where all dissolved P was adsorbed and retained by the ferrihydrite or the transformation products.^16^ Also when phosphate-adsorbed Fe (oxyhydr)oxides (ferrihydrite, goethite, hematite) were coated onto quartz sand and incubated in redox-dynamic temperate forest soils, phosphate was retained by the Fe-mineral phases.^62^ Supported by these similar findings, our results suggest that phosphate can be retained by ferrihydrite despite the exposure to Fe-reducing conditions.

### The Effects of Mixing Minerals with Soil

The effect of the close association between minerals and the soil matrix was reflected in the extent of mineral transformation, which was mineral specific. While the incubation of ^NA^Fe-Fh and ^57^Fe-Fh–soil mixes both resulted in goethite formation, the fraction of goethite at 16 weeks was much higher in ^NA^Fe-Fh mesh bags without soil (82–85%, Figures 2A and S14A) compared to ^57^Fe-Fh–soil mixes (32% of ^57^Fe, Figure 3A). Additionally, ferrihydrite transformation was faster when the ferrihydrite was spatially separated from the soil (66% goethite in ^NA^Fe-Fh at 8 weeks) compared to mineral–soil mixes (^57^Fe-Fh; 9% of ^57^Fe present as goethite at 8 weeks), as indicated by Mössbauer spectroscopy results (measured at 77K; Figures 2 and 3). In contrast, for lepidocrocite, more goethite formed in ^57^Fe-Lp mineral–soil mixes (31% of ^57^Fe present as goethite in 77 K Mössbauer spectra) compared to ^NA^Fe-Lp samples without soil (12% goethite in 77 K Mössbauer spectra; no goethite detected by XRD). Compared to ferrihydrite, lepidocrocite is more stable against mineral transformation, as seen from minor mineral transformations in ^NA^Fe-Lp samples (Figures 2G and S14E). However, when lepidocrocite was mixed with soil, it was likely closely associated with goethite in the soil, which may have facilitated goethite formation from lepidocrocite in this experiment. Additionally, the ^57^Fe-Lp in the soil mixes was directly exposed to the geochemical soil environment that was favorable for goethite formation, as seen from the presence of goethite in the soil. The presence of goethite in the initial ^57^Fe-Lp may have further facilitated goethite formation. This is supported by findings from a Fe(II)-catalyzed mineral transformation study, which found that lepidocrocite was strongly supported when goethite was initially added to the mineral slurries.^29^

The differences between transformation extents of ^NA^Fe-Fh (>80% goethite) and ^NA^Fe-Lp (12% goethite) were large (Figures 2 and S14). This contrasts the similar fractions of goethite that formed from ferrihydrite and lepidocrocite in the ^57^Fe-mineral–soil mixes (32% of remaining ^57^Fe in ^57^Fe-Fh and 31% of remaining ^57^Fe in ^57^Fe-Lp, Figure 3). This comparison suggests that the close association of minerals with the soil may have regulated the mineral transformation extent and products, outweighing effects of initial mineral crystallinity. An explanation for this regulating effect may be that the geochemical conditions inside the mesh bags with ^57^Fe-Fh and ^57^Fe-Lp–soil mixes were likely much more similar to each other and to conditions in the surrounding soil, compared to ^NA^Fe pure mineral mesh bags. This likely directed the trajectory of mineral transformations. For example, during the abiotic oxidation of Fe(II) by oxygen, the mineral products can be impacted by dissolved silicate and phosphate^35,63^ or pre-existing Fe-minerals.^48^ Thus, it is possible that ferrihydrite and lepidocrocite transformations to goethite via the dissolution–reprecipitation pathway^20,64^ in the ^57^Fe-mineral–soil mixes have been similarly affected by these factors.

Mixing the Fe-minerals with soil strongly promoted their reductive dissolution through microbial Fe reduction. For example, for all incubated ^57^Fe-mineral–soil mixes, the solid-associated Fe(II) fractions of the remaining ^57^Fe at 16 weeks were larger compared to ^NA^Fe-mineral mesh bags without soil; 43 vs 0% for Fh, 33 vs 8% for FhP, and 26% vs 6% for Lp (compare Figures 2 with 3). Since part of the ^57^Fe(II) diffused out of the mesh bags, we assume that the reductively dissolved fractions of ^57^Fe-Fh and ^57^Fe-Lp were even larger. The enhanced reductive dissolution in the ^57^Fe-mineral–soil mixes likely occurred due to the immediate physical exposure of minerals to the soil matrix, including Fe reducing bacteria. This demonstrates that in soils, the reductive dissolution of Fe-minerals can compete with their transformation to more crystalline Fe-minerals. This competition results in lower extents of mineral transformation compared to Fe(II)-catalyzed transformations in mineral slurries.

## Environmental Implications

The results of this study demonstrate that mineral transformations of ferrihydrite and lepidocrocite in soils under field conditions differ from those observed in mineral or soil slurries and in flooded soil microcosms in the laboratory. Based on our study, we conclude that Fe-mineral transformations in the field generally occur more slowly and result in lower extents of mineral transformation, compared to laboratory experiments. This may impact the distribution of Fe in soils. In the field, soils are open systems in which advective flow and physical heterogeneity at the pore and aggregate scales affect the local element concentrations in porewater more than in a closed microcosm filled with homogenized soil material. For example, the local buildup of Fe(II) at anoxic microsites in field soils may be different than in microcosms filled with homogenized soils or in mixed soil slurries, potentially altering the composition of mineral transformation products. By comparing the transformations of Fe-minerals in mesh bags in pure form and when mixed with soil, we show that the intimate contact of the Fe-minerals with other soil components (minerals, organic matter, and microorganisms), as it naturally occurs in soils, drastically affects reductive dissolution and mineral transformations. This was seen in large differences between ferrihydrite and lepidocrocite transformation to goethite in the pure-mineral mesh bags, compared to similar transformation extents when the minerals were directly exposed to the soil matrix. Further, we show that adsorbed phosphate strongly hinders Fe-mineral transformations, while it may promote their reductive dissolution. The impact of phosphate on the reductive dissolution versus transformation of Fe-minerals should be considered in rice paddy soils where phosphate fertilizers are used. Further, rice paddy soils are rich in easily reducible and short-range-ordered Fe oxyhydroxides which form as iron plaque around rice roots and during oxic periods, e.g., when the soil is intermittently irrigated or drained for rice harvest. An enhancement of microbial Fe reduction would promote the release of other Fe-mineral-associated nutrients and contaminants to the soil porewater, increasing their potential for the uptake by rice plants. The findings of this study contribute to a better understanding of Fe oxyhydroxide dynamics in reducing soils under field conditions and how these can be affected by phosphate.

## Supporting Information Available

Details on mineral synthesis, experimental and analytical methods; characterization of the minerals by XRD and Mössbauer spectroscopy; characterization of the soil by XRF and Mössbauer spectroscopy; additional porewater data; additional Mössbauer spectra and fitting parameters; X-ray diffraction data of incubated ^NA^Fe-minerals and fitting parameters; element contents in incubated ^NA^Fe-minerals; Aqua regia digestion data (PDF)
