# Supplementary material for: Iron Oxyhydroxide Transformation in a Flooded Rice Paddy Field and the Effect of Adsorbed Phosphate
Source: Environ Sci Technol. 2024 Jun 4;58(24):10601–10. doi: 10.1021/acs.est.4c01519 (PMC11191587; doi:10.1021/acs.est.4c01519)
Supplement: Supplementary file 1 — es4c01519_si_001.pdf [file es4c01519_si_001.pdf]

Supporting Information

Iron oxyhydroxide transformation in a flooded rice paddy field and the effect of adsorbed phosphate

Katrin Schulz[1], Worachart Wisawapipat[2], Kurt Barmettler[1], Andrew R. C. Grigg[1], L. Joëlle Kubeneck[1], Luiza Notini[1], Laurel K. ThomasArrigo[1,3,*], and Ruben Kretzschmar[1,*]

[1] *Soil Chemistry Group, Institute of Biogeochemistry and Pollutant Dynamics, CHN, ETH Zurich, 8092 Zurich, Switzerland*

[2] *Department of Soil Science, Faculty of Agriculture, Kasetsart University, Bangkok 10900, Thailand*

[3] *Environmental Chemistry Group, Institute of Chemistry, University of Neuchâtel, 2000 Neuchâtel, Switzerland (current address)*

**Correspondence: laurel.thomas@unine.ch and ruben.kretzschmar@env.ethz.ch*

(contains 17 figures and 7 tables)

Table of Contents

S1. Soil characterization 2
S2. Initial mineral characterization 4
S3. Experimental setup 7
S4. Soil redox potentials 8
S5. Porewater element concentrations 8
S6. Mössbauer spectroscopy 9
Method details 9
Mineral transformations in ^{NA}Fe mineral samples without soil 10
Mössbauer fitting parameters of ^{NA}Fe mineral samples 12
Mineral transformations in ^{57}Fe-mineral-soil mixes 15
Mössbauer fitting parameters of ^{57}Fe-mineral-soil mixes 17
S7. X-ray diffraction 20
Method details 20
Mineral transformations in ^{NA}Fe-mineral mesh bags without soil 21
Fitting parameters of Rietveld quantitative phase analysis 22
S8. Element contents in incubated ^{NA}Fe minerals 23
S9. Aqua regia digestion results 23
References 26

S1. Soil characterization

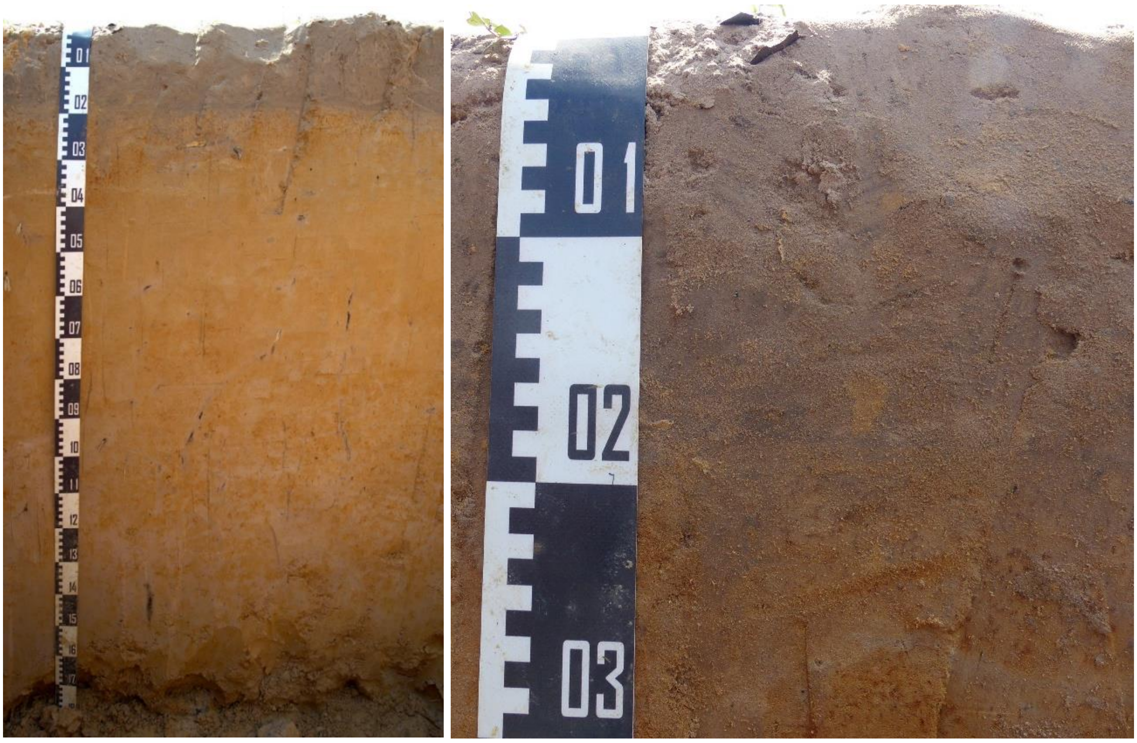

Figure S1: Soil profile at the Ubon Ratchathani Rice Research Center, Thailand, February 2020. Images show the soil profile across the full depth (left; 0-180 cm) and the soil at the depth where samples were installed in June 2021 (right; 10-15 cm).

The soil samples were taken during the dry season in February 2020 at the Ubon Ratchathani Rice Research Center (URRC) in Thailand. A soil profile of 2 m depth (Figure S1) was established at the sampling site for soil characterization. The soil was classified as a Hydragric Loamic Anthrosol on sandstone after the World Reference Base for Soil Resources.[1] The puddled horizon was 20 cm thick, with a dense and hard plough pan at 20 cm (anthraquic horizon). Beneath the plough pan, prominent orange-brown hydromorphic features were present (hydragric horizons). While hydromorphic features were sharp and small, and partly followed plant root channels in the horizon beneath the plough pan (20-30 cm), the features became larger and blurred at greater depths (30-130 cm). Below the groundwater table (>130 cm), the soil had a bleached color and only showed few hydromorphic features.

Table S1: Characterization of the experimental rice paddy soil (0-15 cm depth), with element concentrations measured in dried, sieved (<2 mm) and milled soil by X-ray fluorescence spectroscopy (XRF), an elemental analyzer (EA), or after total digestion with hydrofluoric acid (HF) and the texture determined on dried and sieved (<2 mm) soil. These values have been reported previously in Schulz et al.[2]

**Element concentrations**	Fe	3.3 g kg^{-1}	XRF
	Si	417.4 g kg^{-1}	XRF
	Al	14.0 g kg^{-1}	XRF
	C	4.0 g kg^{-1}	EA
	N	0.5 g kg^{-1}	EA
	P	0.08 g kg^{-1}	HF
**Texture**	Sand	84.8%	
	Silt	12.6%	
	Clay	2.6%	

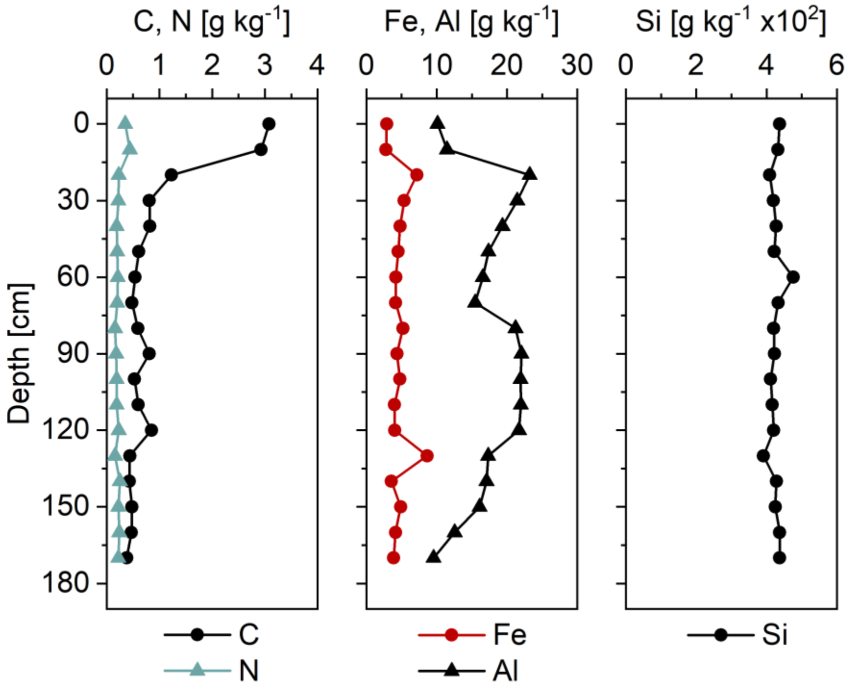

Figure S2: Depth profiles of solid element contents of carbon (C), nitrogen (N), iron (Fe), aluminum (Al) and silicon (Si) determined by X-ray fluorescence spectroscopy in samples from the soil profile established in the experimental rice paddy field during the sampling campaign in the dry season in February 2020 at the Ubon Ratchathani Rice Research Center, Thailand.

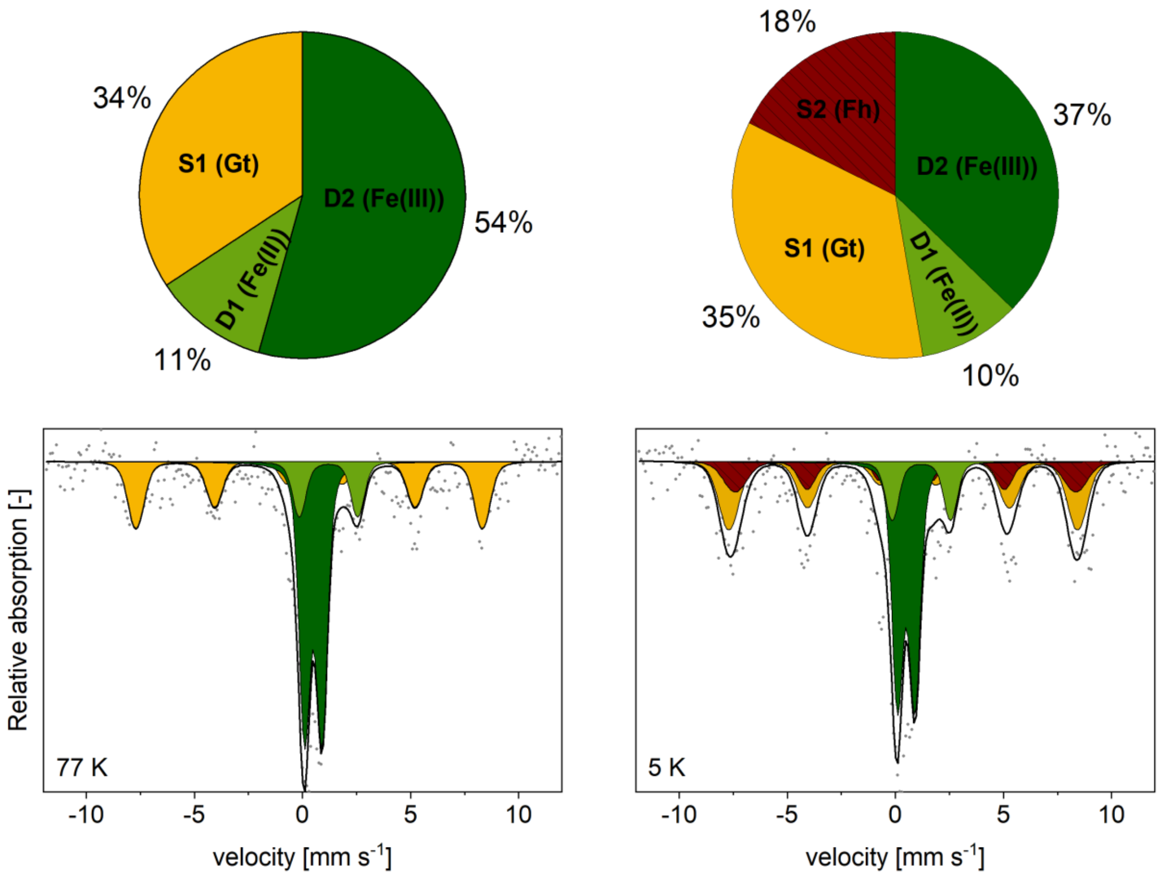

Figure S3: Mössbauer spectra collected at 77 K (left) and 5 K (right) from of the rice paddy soil sampled in February 2020 during the dry season. Respective spectral areas of fitted mineral fractions are given in pie charts. The colors and labels in the pie charts correspond to the colors of the fitting components in the Mössbauer spectra. Abbreviations: Fh = ferrihydrite, Gt = goethite, D = doublet, S = sextet. This data has been reported previously in Schulz et al.[2]

S2. Mineral synthesis and characterization

For ferrihydrite (^{NA}Fe-Fh) synthesis a 0.2 M ferric (Fe(III)) nitrate solution (~pH 1) was titrated (836 Titrando, Metrohm) with 1 M NaOH (NORMATOM®, Merck) to pH 7.5±0.1 under vigorous stirring at room temperature. For lepidocrocite (^{NA}Fe-Lp) synthesis, a 0.2 M ferrous (Fe(II)) chloride solution (300 mL) was titrated to pH 6.7-6.9 with 1 M NaOH at room temperature and then oxidized under vigorous stirring and gentle purging with air (approx. 100 mL min^{-1}). During the oxidation, the further addition of 1 M NaOH was required to maintain the pH at 6.7-6.9. The isotopically labeled Fe(II) solutions for the ^{57}Fe-labeled ferrihydrite (^{57}Fe-Fh) and lepidocrocite (^{57}Fe-Lp) synthesis were prepared by dissolving ^{57}Fe(0) (96.14% ^{57}Fe, Isoflex USA) in 2 M HCl (NORMATOM®, 34-37 %, VWR) overnight. For ^{57}Fe-Fh synthesis, the Fe(II) solution was oxidized to Fe(III) for one hour with excess H_2O_2 (35%, Merck). The Fe(II) and Fe(III) solutions were passed through a 0.45 µm nylon filter, and

minerals were synthesized as described for ^{NA}Fe-Fh and ^{NA}Fe-Lp. All precipitates were repeatedly washed in ultra-pure water (UPW, >18.2 MΩ·cm, Milli-Q, Merck Millipore), centrifuged (3800 g for 15 min, 20 °C) and the supernatants were decanted. All precipitates were resuspended in UPW, shock frozen dropwise in liquid N_2, freeze dried and gently homogenized with a mortar and pestle. The dried mineral powders were stored in amber glass bottles in a desiccator.

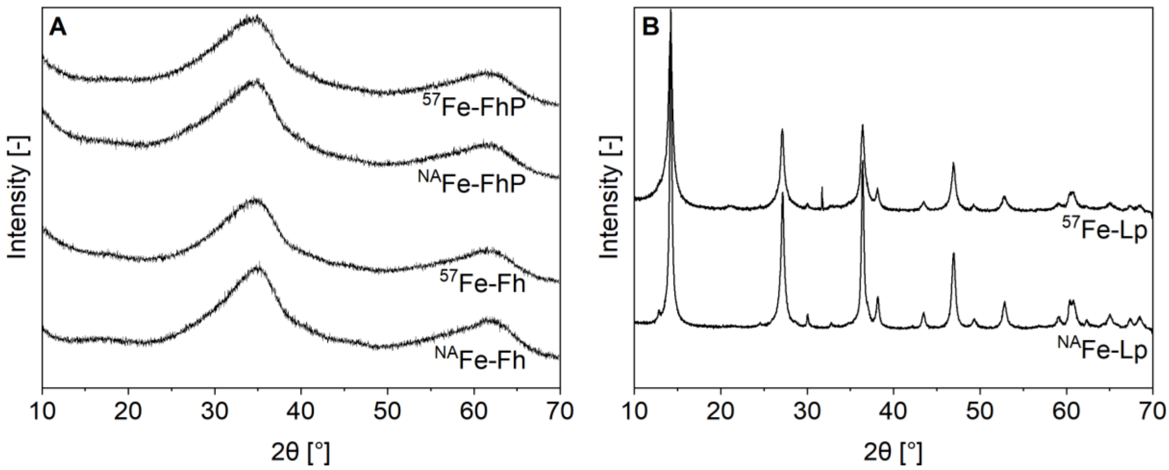

Figure S4: X-ray diffraction patterns of initial ferrihydrites (Fh) and phosphate-adsorbed ferrihydrites (FhP) (A) and lepidocrocites (B) with natural abundance Fe (^{NA}Fe-Fh, ^{NA}Fe-FhP, ^{NA}Fe-Lp) and isotopically labeled with ^{57}Fe (^{57}Fe-Fh, ^{57}Fe-FhP, ^{57}Fe-Lp).

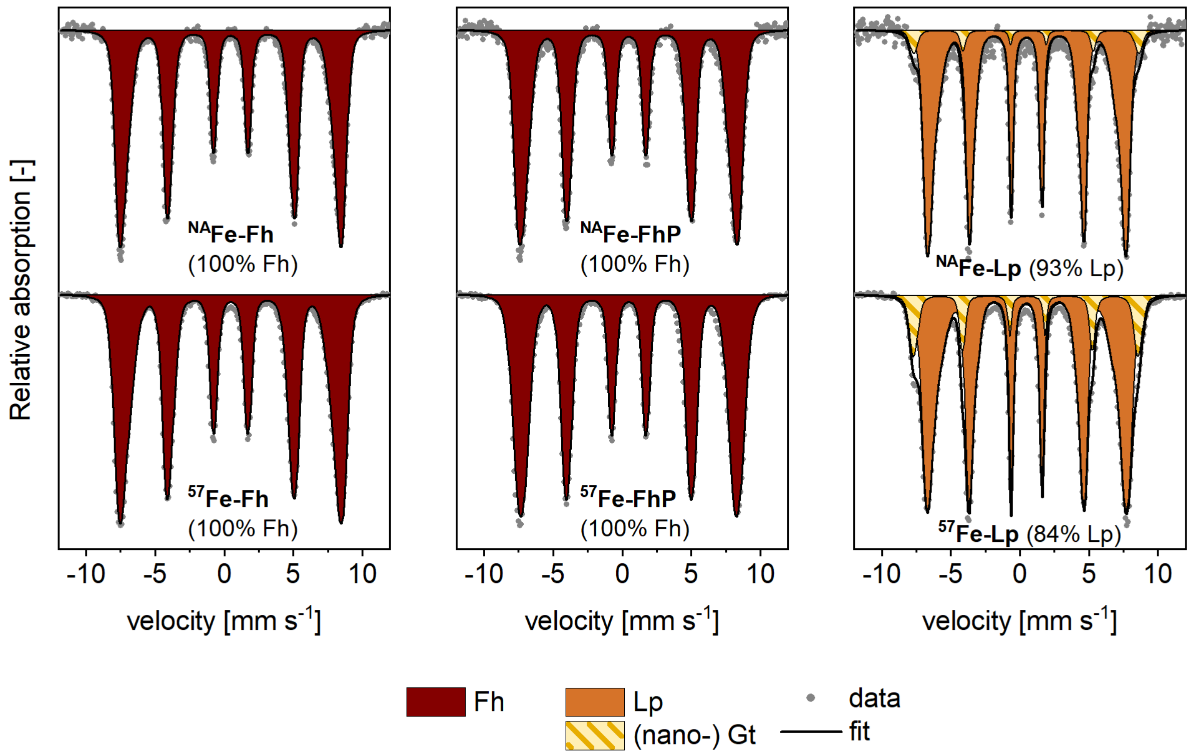

Figure S5: Mössbauer spectra (5 K) of the initial ^{NA}Fe-minerals (top panel) and ^{57}Fe-mineral-soil mixes (bottom panel). Spectra of ferrihydrite (Fh; left), ferrihydrite with adsorbed P (FhP; middle), and lepidocrocite (Lp; right) are displayed. Fitting parameters are presented in Table S5. Abbreviations: Fh = ferrihydrite, Lp = lepidocrocite, Gt = goethite.

S3. Experimental setup

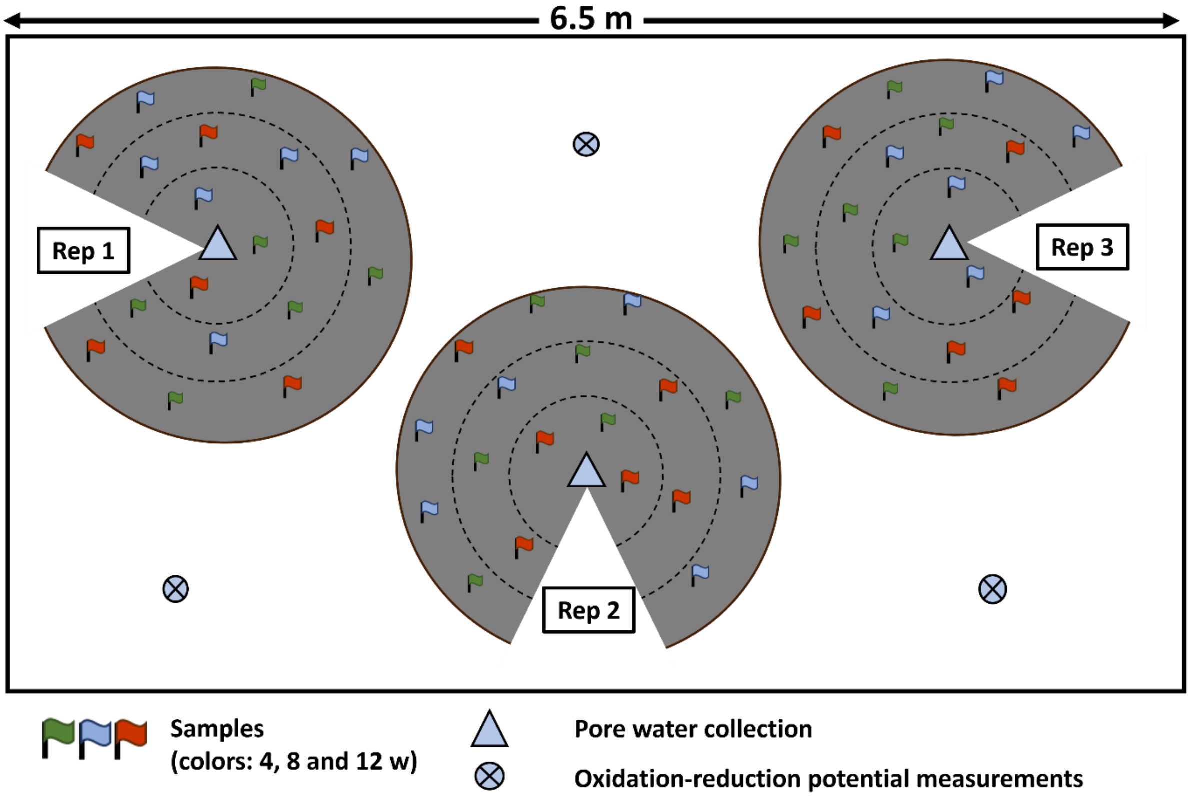

Figure S6: Experimental setup of field experiment with three replicate (Rep) setups. Abbreviations: w = weeks.

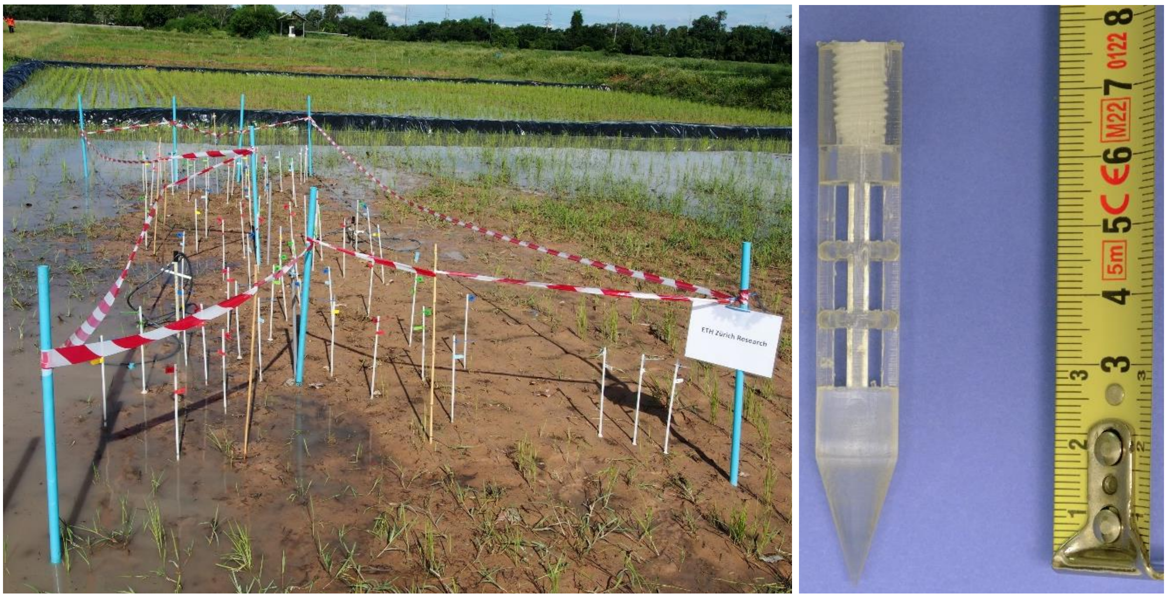

Figure S7: Photo of the experimental setup in at the Ubon Ratchathani Rice Research Center, Thailand, in June 2021, and an empty sample holder.

S4. Soil redox potentials

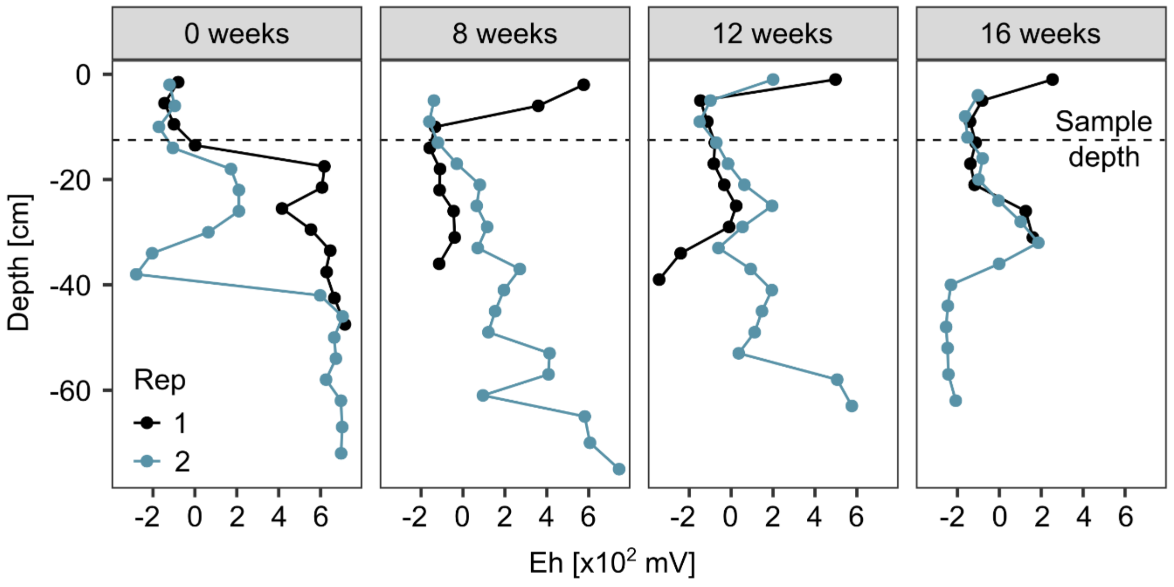

Figure S8: Soil redox potentials (Eh) on the day of the experimental setup (0 weeks) and at sampling days after 8, 12 and 16 weeks. The depth where mineral samples were installed is marked by the dashed line. Replicate measurements (Rep) are indicated by colors.

S5. Porewater element concentrations

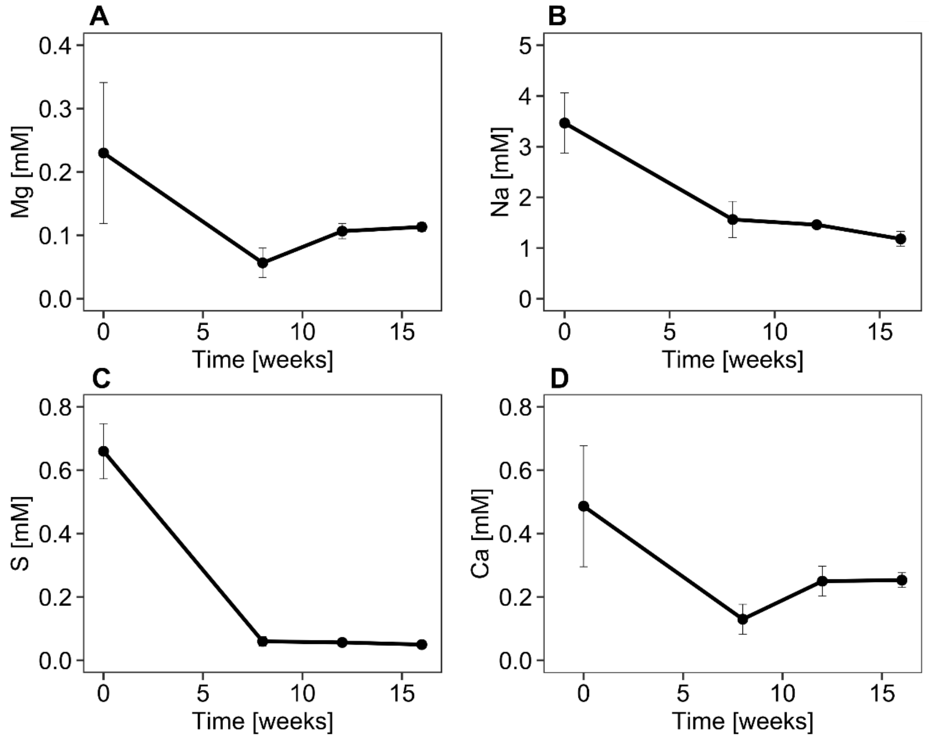

Figure S9: Aqueous element concentrations of magnesium (A), sodium (B), total sulfur (C) and calcium (D) in soil porewaters at the start and during the experiment.

S6. Mössbauer spectroscopy

Method details

For the preparation of Mössbauer samples for ^{NA}Fe minerals without soil, triplicate samples were combined and ~15 mg of mineral was suspended in 1.5 mL UPW and deposited on a 0.22 µm PVDF filter. The sample was dried in the glovebox and sealed in two layers of Kapton® tape. For ^{57}Fe mineral-soil mixes, Mössbauer samples were prepared by combining triplicate samples and by sealing representative aliquots (~150 mg) of the dry mineral-soil mixes in two layers of Kapton® tape.

All samples were prepared anoxically and stored in the glovebox until analysis. The Mössbauer spectra were collected at 77 K and 5 K using a closed-cycle He cryostat (SHI-850, Janis Research Co.). The spectrometer was equipped with a ^{57}Co source in a standard setup (WissEl, Wissenschaftliche Elektronik GmbH) and a 7 µm thick α-Fe(0) foil was used for calibration at room temperature. The spectra were analyzed and quantitatively interpreted using the Recoil software (ref [3] ; Ottawa, Canada) by applying an extended Voigt-based fitting routine, with the half-width at half-maximum fixed to 0.135 mm s^{-1}, which equals to the inner line broadening of the calibration foil.

Mineral transformations in ^{NA}Fe mineral samples without soil

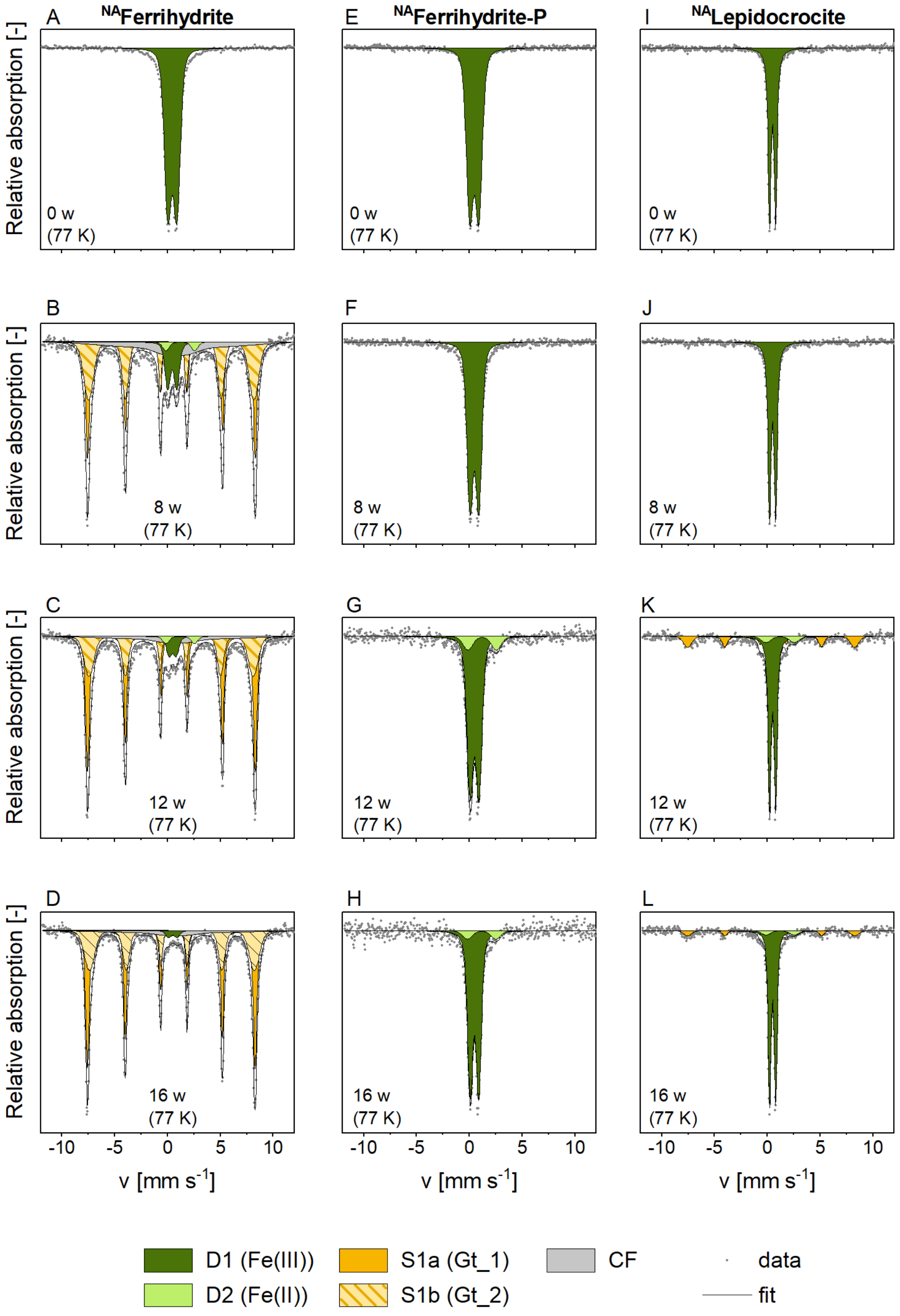

Figure S10: Mössbauer spectra collected at 77 K of initial and reacted ^{NA}Fe minerals. Abbreviations: w = weeks, Gt = goethite, CF = collapsed feature. Fitting parameters are presented in Table S2.

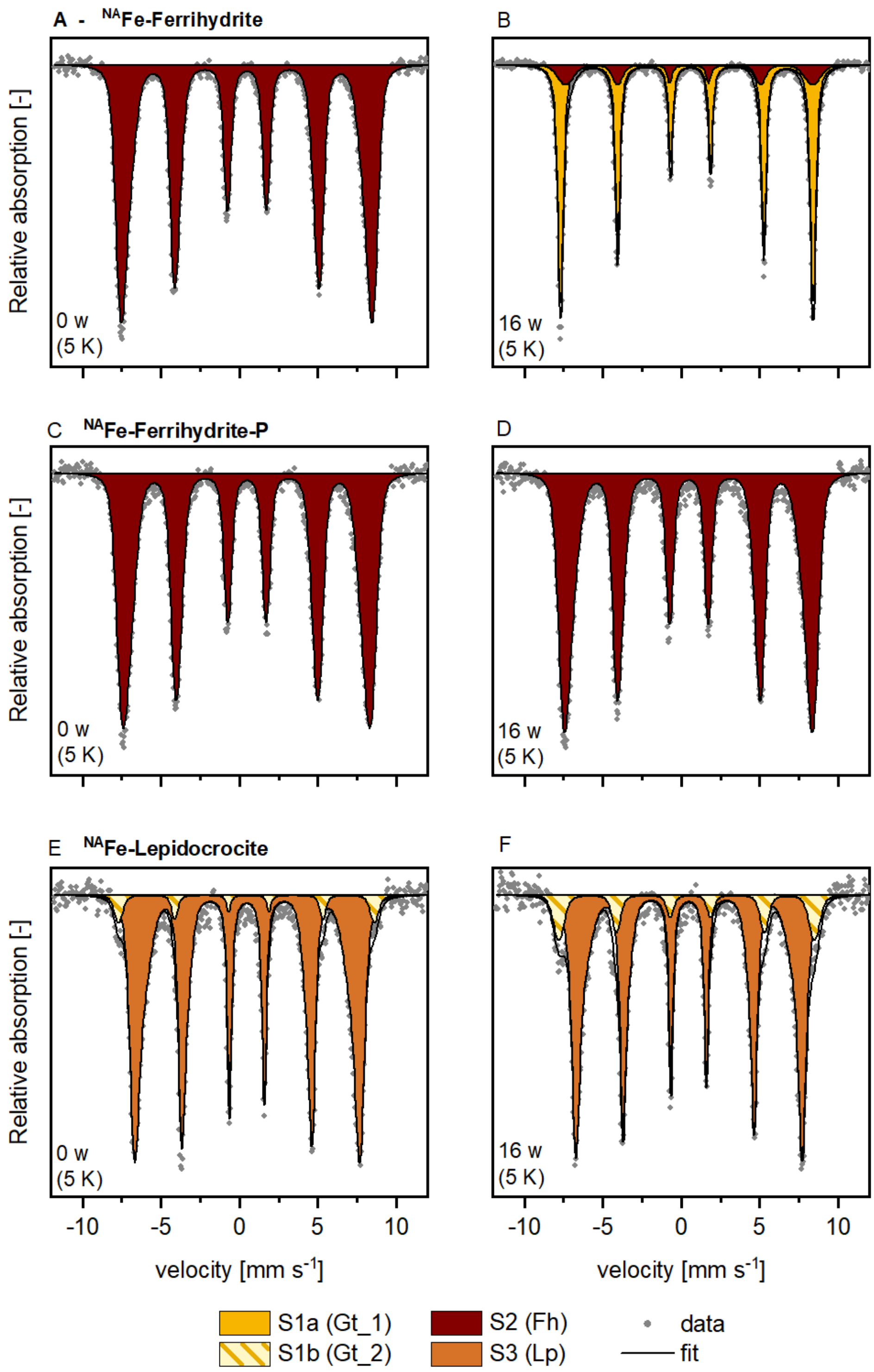

Figure S11: Mössbauer spectra collected at 5 K of initial (0 w) and incubated (16 w) ^{NA}Fe minerals. Abbreviations: w = weeks, Fh = ferrihydrite, Lp = lepidocrocite, Gt = goethite. Fitting parameters are presented in Table S3.

Mössbauer fitting parameters of ^{NA}Fe mineral samples

Table S2: Mössbauer fitting parameters for spectra collected at 77 K from initial and incubated ^{NA}Fe-ferrihydrite (^{NA}Fe-Fh), ^{NA}Fe-ferrihydrite-P (^{NA}Fe-FhP) and ^{NA}Fe-lepidocrocite (^{NA}Fe-Lp) samples without soil.

**Sample**	**Time [w]**	**Component**	**CS [a] [mm s^{-1}]**	**QS [b] or ε [c] [mm s^{-1}]**	**σ_{QS} or σ_{ε} [d] [mm s^{-1}]**	**H [e] [T]**	**σ_H [d] [T]**	**Area [%]**	**Red. χ^2 [f]**
**^{NA}Fe-Fh (77 K)**	0	D1 – Fe(II)	-	-	-	-	-	0	4.89
		D2 – Fe(III)	0.45	0.88	0.55	-	-	100	
		S1a – Gt_1	-	-	-			0	
		S1b – Gt_2	-	-	-			0	
		CF	-	-	-			0	
	8	D1 – Fe(II)	-	-	-	-	-	0	1.21
		D2 – Fe(III)	0.45	0.86	0.50*	-	-	8.6	
		S1a – Gt_1	0.50	-0.13	-	49.21	0.54	30.2	
		S1b – Gt_2	0.45	-0.09	48.53	2.50*	0.45	35.7	
		CF	0.80*	-	46.67*	31.97*	0.80*	25.5	
	12	D1 – Fe(II)	1.20*	2.70*	0.50*	-	-	2	1.52
		D2 – Fe(III)	0.49	0.48	0.40*	-	-	4.4	
		S1a – Gt_1	0.49	-0.13	-	49.20	0.66	47.0	
		S1b – Gt_2	0.43	-0.09	-	49.19	2.50*	30.8	
		CF	0.80*	-	46.67*	31.97*	0.80*	15.8	
	16	D1 – Fe(II)	-	-	-	-	-	0	1.80
		D2 – Fe(III)	0.50*	0.80*	0.39*	-	-	1.8	
		S1a – Gt_1	0.47	-0.12	-	49.20	0.59	50.2	
		S1b – Gt_2	0.51	-0.10*	-	48.39	2.64	35.2	
		CF	0.80*	-	46.67*	31.97*	0.80*	12.8	

Table S2 continued

Sample	Time [w]	Component	CS [a] [mm s^{-1}]	QS [b] or ε [c] [mm s^{-1}]	σ_{QS} or σ_{ε} [d] [mm s^{-1}]	H [e] [T]	σ_H [d] [T]	Area [%]	Red. χ^2 [f]
**^{NA}Fe-FhP (77 K)**	0	D1 – Fe(II)	-	-	-	-	-	0	1.53
		D2 – Fe(III)	0.47	0.80	0.37	-	-	100	
	8	D1 – Fe(II)	-	-	-	-	-	0	1.65
		D2 – Fe(III)	0.47	0.85	0.47	-	-	100	
	12	D1 – Fe(II)	1.2*	2.7*	0.5*	-	-	11.5	0.85
		D2 – Fe(III)	0.48	0.86	0.45	-	-	88.5	
	16	D1 – Fe(II)	1.20*	2.70*	0.50*	-	-	8.1	0.65
		D2 – Fe(III)	0.47	0.81	0.37	-	-	91.9	
**^{NA}Fe-Lp (77 K)**	0	D1 – Fe(II)	-	-	-	-	-	0	0.82
		D2 – Fe(III)	0.49	0.58	0.12	-	-	100	
		S1a – Gt_1	-	-	-	-	-	0	
	8	D1 – Fe(II)	-	-	-	-	-	0	1.44
		D2 – Fe(III)	0.49	0.58	0.14	-	-	100	
		S1a – Gt_1	-	-	-	-	-	0	
	12	D1 – Fe(II)	1.20*	2.70*	0.50*	-	-	7.2	1.14
		D2 – Fe(III)	0.49	0.58	0.12	-	-	71.5	
		S1a – Gt_1	0.45	-0.1*	-	49.00*	2.00*	21.4	
	16	D1 – Fe(II)	1.20*	2.70*	0.50*	-	-	5.8	0.81
		D2 – Fe(III)	0.49	0.58	0.19	-	-	82.7	
		S1a – Gt_1	0.45*	-0.10*	0	49.02	2.04	11.6	

[a] Center shift;
[b] Quadrupole splitting (for doublets);
[c] Quadrupole shift (for sextets);
[d] σ, standard deviation of QS, ε or H;
[e] Hyperfine field;
[f] Red. χ^2, goodness of fit;
* Indicates values that were fixed during the fitting process.
Abbreviations: w = week, Gt = goethite, CF = collapsed feature, D = doublet, S = sextet.

Table S3: Mössbauer fitting parameters for spectra collected at 5 K from initial and incubated ^{NA}Fe-ferrihydrite (^{NA}Fe-Fh), ^{NA}Fe-ferrihydrite-P (^{NA}Fe-FhP) and ^{NA}Fe-lepidocrocite (^{NA}Fe-Lp) samples without soil. For FhP and Lp a fitting approach with two components for the hyperfine field (H_1, H_2) was used.

Sample	Time [w]	Component	CS [a] [mm s^{-1}]	ε [b] [mm s^{-1}]	σ_ε [c] [mm s^{-1}]	H_1 [d] [T]	σ_{H1} [c] [T]	H_2 [d] [T]	σ_{H2} [c] [T]	Frac. H_2 [-]	Area [%]	Red. χ^2 [e]
**^{NA}Fe-Fh**	0	S2 – Fh	0.48	-0.004	0.16	48.80	2.70	-	-	-	100	1.52
	16	S1a – Gt_1	0.48	-0.12	-	49.89	0.61				84.1	
		S2 – Fh	0.48	-0.004	0.16	48.80	2.70				15.9	3.6
**^{NA}Fe-FhP**	0	S2 – Fh	0.47	-0.01	0.17	49.35	1.47	46.0*	2.00*	0.42	100	1.09
	16	S2 – Fh	0.47	-0.01	0.17	49.35	1.51	46.2	2.57	0.40	100	0.91
**^{NA}Fe-Lp**	0	S3 – Lp	0.49	0.01	-	44.6	1.33	41.79	3.14	0.47	93.3	1.17
		S1b – Gt_2	0.53	-0.07	-	50.55	1.28	-	-	-	6.7	
	16	S3 – Lp	0.49	0.02	-	44.9	1.00	42.5	2.64	0.45	82.9	0.91
		S1b – Gt_2	0.47	-0.04	0	50.74	1.51	-	-	-	17.1	

[a] Center shift;
[b] Quadrupole shift;
[c] σ, standard deviation of ε or H;
[d] Hyperfine field (fitted with two components H_1 and H_2);
[e] Red. χ^2, goodness of fit;
* Indicates values that were fixed during the fitting process.
Abbreviations: w = week, Fh = ferrihydrite, Gt = goethite, Lp = lepidocrocite, D = doublet, S = sextet.

Mineral transformations in ^{57}Fe-mineral-soil mixes

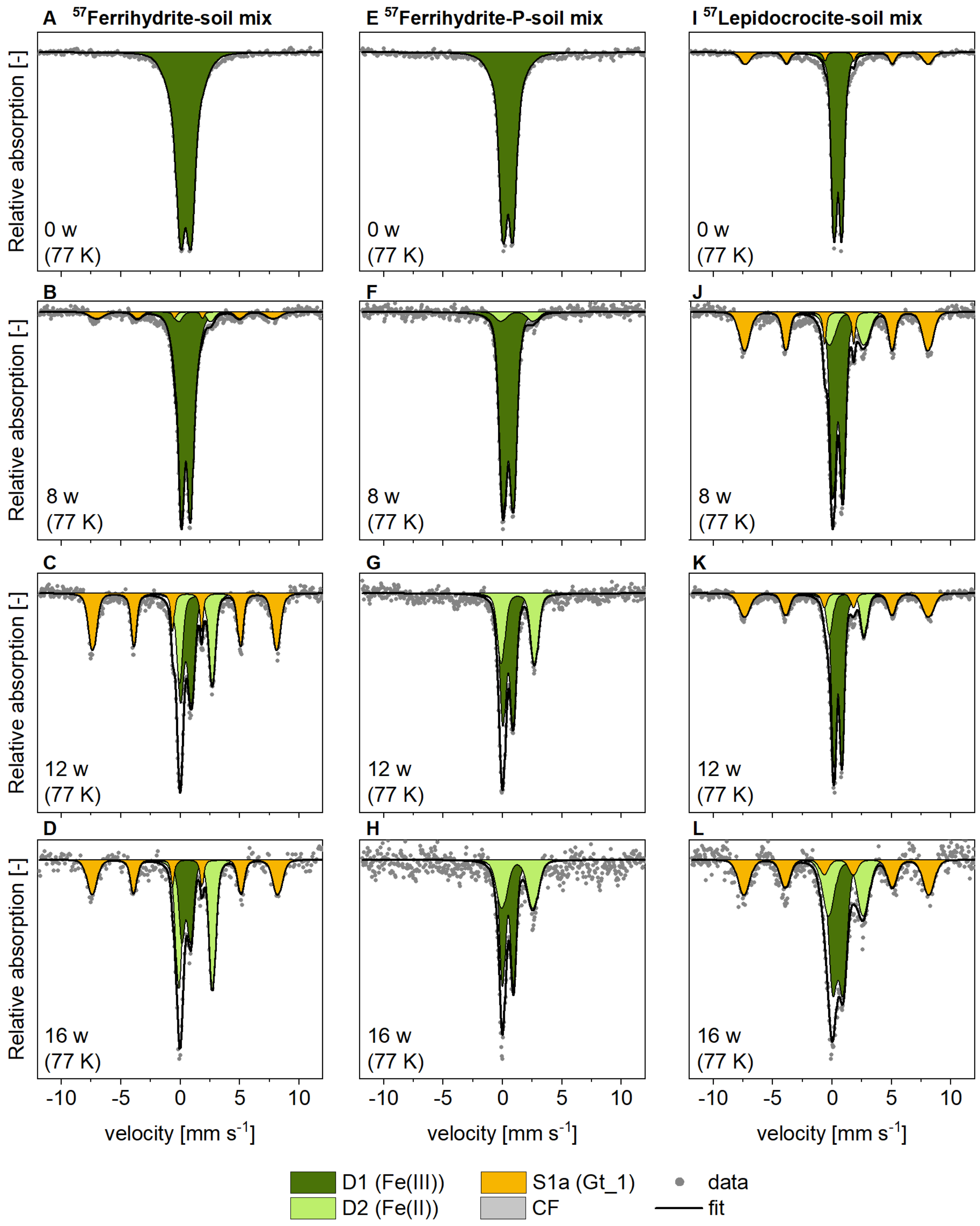

Figure S12: Mössbauer spectra collected at 77 K from ^{57}Fe-mineral-soil mixes. Abbreviations: w = weeks, Gt = goethite, CF = collapsed feature. Fitting parameters are presented in Table S4.

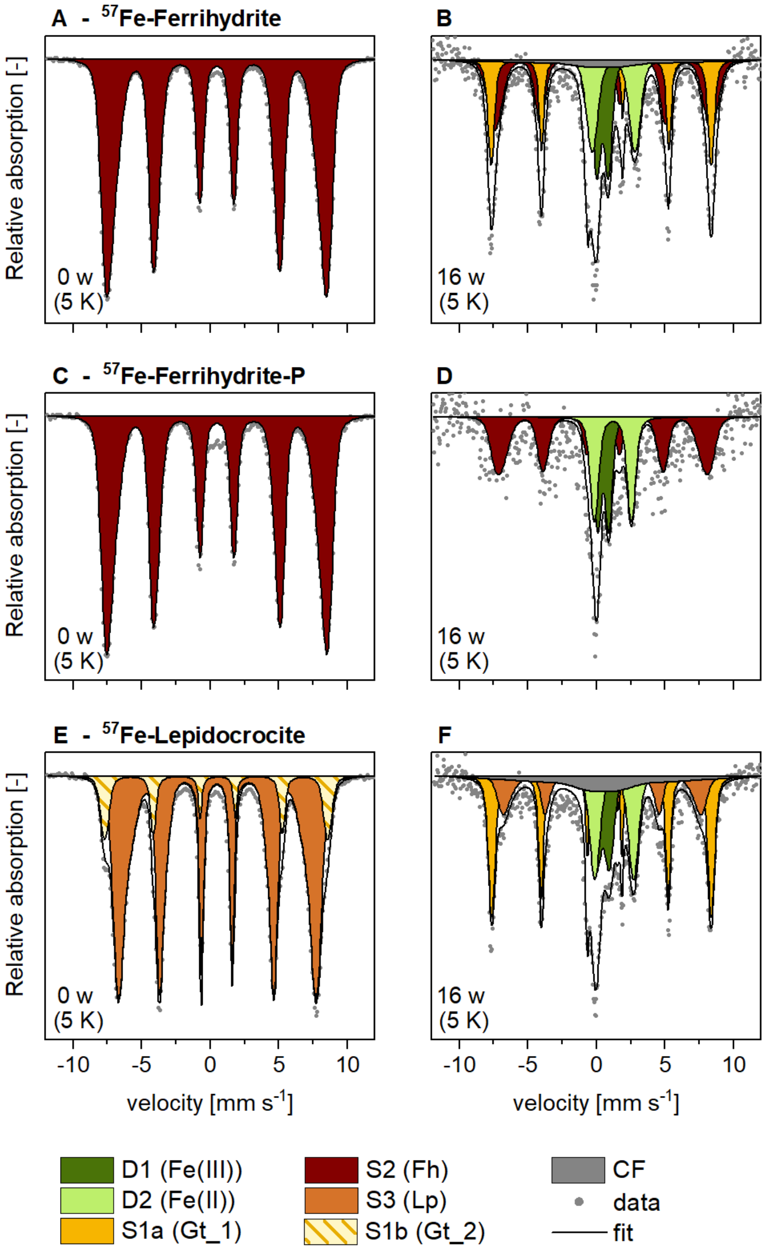

Figure S13: Mössbauer spectra collected at 5 K of initial (0 w) and incubated (16 w) ^{57}Fe mineral-soil mixes. Abbreviations: w = weeks, Fh = ferrihydrite, Lp = lepidocrocite, Gt = goethite, CF = Collapsed feature. Fitting parameters are presented in Table S5.

Mössbauer fitting parameters of ^{57}Fe-mineral-soil mixes

Table S4: Mössbauer fitting parameters for spectra collected at 77 K from initial and incubated ^{57}Fe-ferrihydrite-soil mixes (^{57}Fe-Fh), ^{57}Fe-ferrihydrite-P-soil mixes (^{57}Fe-FhP) and ^{57}Fe-lepidocrocite-soil mixes (^{57}Fe-Lp).

Sample	Time [w]	Component	CS [a] [mm s^{-1}]	QS_1 [b] or ε_1 [c] [mm s^{-1}]	σ_{QS1} or $\sigma_{\varepsilon1}$ [d] [mm s^{-1}]	QS_2 [b] or ε_2 [c] [mm s^{-1}]	σ_{QS2} or $\sigma_{\varepsilon2}$ [d] [mm s^{-1}]	Frac QS_2 or ε_2 [-]	H [e] [T]	σ_H [d] [T]	Area [%]	Red. χ^2 [f]
**^{57}Fe-Fh (77 K)**	0	D1 – Fe(III)	0.45	0.86	0.52	1.00*	2.00*	0.32	-	-	100.0	0.84
	8	D1 – Fe(III)	0.47	0.79	0.39	1.00*	2.00*	0.31	-	-	89.0	1.14
		D2 – Fe(II)	1.20*	2.70*	0.50*	-	-	-	-	-	1.6	
		S1a – Gt_1	0.533	-0.10	-	-	-	-	47.00*	3.00*	9.4	
	12	D1 – Fe(III)	0.47	0.91	0.44	-	-	-	-	-	32.8	0.94
		D2 – Fe(II)	1.30	2.75	0.42	-	-	-	-	-	27.0	
		S1a – Gt_1	0.47	-0.11	-	-	-	-	47.99	1.67	40.3	
	16	D1 – Fe(III)	0.50	0.75	0.37	-	-	-	-	-	25.1	0.70
		D2 – Fe(II)	1.29	2.84	0.40	-	-	-	-	-	42.7	
		S1a – Gt_1	0.49	-0.10	-	-	-	-	48.39	2.06	32.0	
**^{57}Fe-FhP (77 K)**	0	D1 – Fe(III)	0.47	0.81	0.42	-	-	-	-	-	100.0	1.89
	8	D1 – Fe(III)	0.48	0.88	0.47	-	-	-	-	-	93.4	0.62
		D2 – Fe(II)	1.20*	2.70*	0.50*	-	-	-	-	-	6.6	
	12	D1 – Fe(III)	0.46	0.86	0.4	-	-	-	-	-	60.8	0.82
		D2 – Fe(II)	1.29	2.79	0.52	-	-	-	-	-	39.2	
	16	D1 – Fe(III)	0.43	0.87	0.42	-	-	-	-	-	67.5	0.44
		D2 – Fe(II)	1.29	2.8	0.37	-	-	-	-	-	32.5	

Table 4 continued

Sample	Time [w]	Component	CS [a] [mm s^{-1}]	QS$_1$ [b] or ε$_1$ [c] [mm s^{-1}]	σ$_{QS1}$ or σ$_{ε1}$ [d] [mm s^{-1}]	QS$_2$ [b] or ε$_2$ [c] [mm s^{-1}]	σ$_{QS2}$ or σ$_{ε2}$ [d] [mm s^{-1}]	Frac QS$_2$ or ε$_2$ [-]	H [e] [T]	σ$_H$ [d] [T]	Area [%]	Red. χ^2 [f]
**^{57}Fe-Lp (77 K)**	0	D1 – Fe(III)	0.49	0.64	0.32	-	-	-	-	-	95.0	1.05
		S1a- Gt_1	0.50	-0.11	-	-	-	-	47.66	2.00*	15.0	
	8	D1 – Fe(III)	0.48	0.84	0.38	-	-	-	-	-	50.1	1.93
		D2 – Fe(II)	1.20	2.88	0.86	-	-	-	-	-	16.1	
		S1a – Gt_1	0.47	-0.12	-	-	-	-	47.85	2.3	33.8	
	12	D1 – Fe(III)	0.49	0.66	0.26	-	-	-	-	-	52.7	1.61
		D2 – Fe(II)	1.23	2.91	0.42	-	-	-	-	-	17.3	
		S1a – Gt_1	0.47	-0.10	-	-	-	-	47.93	2.28	30.1	
	16	D1 – Fe(III)	0.49	0.9	0.53	-	-	-	-	-	45.3	0.60
		D2 – Fe(II)	1.16	2.9	0.85	-	-	-	-	-	25.8	
		S1a – Gt_1	0.46	-0.10	-	-	-	-	48.2	1.77	30.8	

[a] Center shift;
[b] Quadrupole splitting (for doublets; fitted with two components QS$_1$ and QS$_2$);
[c] Quadrupole shift (for sextets; fitted with two components ε$_1$ and ε$_2$);
[d] σ, standard deviation of QS, ε or H;
[e] Hyperfine field;
[f] Red. χ^2, goodness of fit;
* Indicates values that were fixed during the fitting process.
Abbreviations: w = week, Gt = goethite, D = doublet, S = sextet.

Table S5: Mössbauer fitting parameters for spectra collected at 5 K from initial and incubated ^{57}Fe-ferrihydrite-soil mixes (^{57}Fe-Fh), ^{57}Fe-ferrihydrite-P-soil mixes (^{57}Fe-FhP) and ^{57}Fe-lepidocrocite-soil mixes (^{57}Fe-Lp).

Sample	Time [w]	Component	CS [a] [mm s^{-1}]	QS [b] or ε [c] [mm s^{-1}]	σ_{QS} or σ_{ε} [d] [mm s^{-1}]	H_1 [e] [T]	σ_{H1} [d] [T]	H_2 [e] [T]	σ_{H2} [d] [T]	Frac. H_2 [-]	Area [%]	Red. χ^2 [f]
**^{57}Fe-Fh (5 K)**	0	S1 – Fh	0.48	-0.003	0.19	50.17	1.55	46.67	2.85	0.49	100.0	11.17
	16	D1 – Fe(III)	0.45*	0.81	0.40	-	-	-	-	-	13.6	0.65
		D2 – Fe(II)	1.25*	3.1	0.60	-	-	-	-	-	15.1	
		S1a – Gt_1	0.49	-0.14	-	49.72	1.10	-	-	-	26.0	
		S2 – Fh	0.47*	-0.01*	0.19*	48.85*	2.74*	-	-	-	30.8	
		CF	0.50*	-		27.02*	17.17*	-	-	-	14.4	
**^{NA}Fe-FhP (5 K)**	0	S1 – Fh	0.48	-0.01	0.13	49.37	1.47	46.00*	2.00*	0.42	100	1.09
	16	D1 – Fe(III)	0.50*	0.80*	0.40*	-	-	-	-	-	24.0	0.84
		D2 – Fe(II)	1.20*	2.70*	0.50*	-	-	-	-	-	26.4	
		S2 – Fh	0.48	-0.004	0.19	49.00*	1.50*	46.00*	2.80*	0.77	49.6	
**^{57}Fe-Lp (5 K)**	0	S1b – Gt_2	0.49	-0.05	-	50.37	1.50*				15.6	
		S3 – Lp	0.49	0.02	-	44.82	2.00*	39.76	3.00*	0.28	84.4	1.43
	16	D1 – Fe(III)	0.47	0.91*	0.55*	-	-	-	-	-	13.7	1.02
		D2 – Fe(II)	1.29	2.83*	0.60*	-	-	-	-	-	17.0	
		S1a – Gt_1	0.48	-0.12	-	49.61	1.14	-	-	-	34.0	
		S3 – Lp	0.42	0.02*	-	45.00*	2.00*	42.00*	3.00*	0.38	16.3	
		CF	0.50*	-	-	46.66*	31.97*	-	-	-	19.0	

[a] Center shift;
[b] Quadrupole splitting (for doublets);
[c] Quadrupole shift (for sextets);
[d] σ, standard deviation of QS, ε or H;
[e] Hyperfine field (fitted with two components H_1 and H_2);
[f] Red. χ^2, goodness of fit;
* Indicates values that were fixed during the fitting process.
Abbreviations: w = week, Fh = ferrihydrite, Gt = goethite, Lp = lepidocrocite, CF = collapsed feature, D = doublet, S = sextet.

S7. X-ray diffraction

Method details

Samples for XRD analysis were prepared by resuspending ~2 mg dried sample material in ~30 µL ethanol and pipetting the suspension onto a zero-background holders (silicon wafer, Sil'tronix Silicon Technologies, France). Samples were measured on an XRD instrument, which was equipped with Cu $K\alpha_1$ and $K\alpha_2$ radiation (λ_1 = 1.540526 Å, λ_1 = 1.544398, 40 kV, 40 mA) and a high-resolution energy-dispersive 1D detector (LYNXEYE). To prevent oxidation of Fe(II), all samples were prepared in the glovebox under nitrogen atmosphere using oxygen-free ethanol. The exposure of the samples to oxygen was minimized by using an airtight specimen holder (Bruker AXS, A100B138-B141) and XRD scans were collected from 10-70° 2θ in increments of 0.02° and an acquisition time of 4 s per step.

The XRD patterns were fitted in TOPAS software (Bruker AXS, Version 5) using Rietveld fitting, to identify and quantify mineral phase fractions. Published structure files from the inorganic crystal structure database (ICSD, FIZ Karlsruhe) were used for lepidocrocite (ICSD 93948)[4] and goethite (ICSD 239321).[5] Ferrihydrite fractions were included in the fit as a mass-calibrated hkl phase, following the partial or no known crystal structure (PONKCS)[6] approach, which has been applied in earlier studies.[7–9]

Mineral transformations in ^{NA}Fe-mineral mesh bags without soil

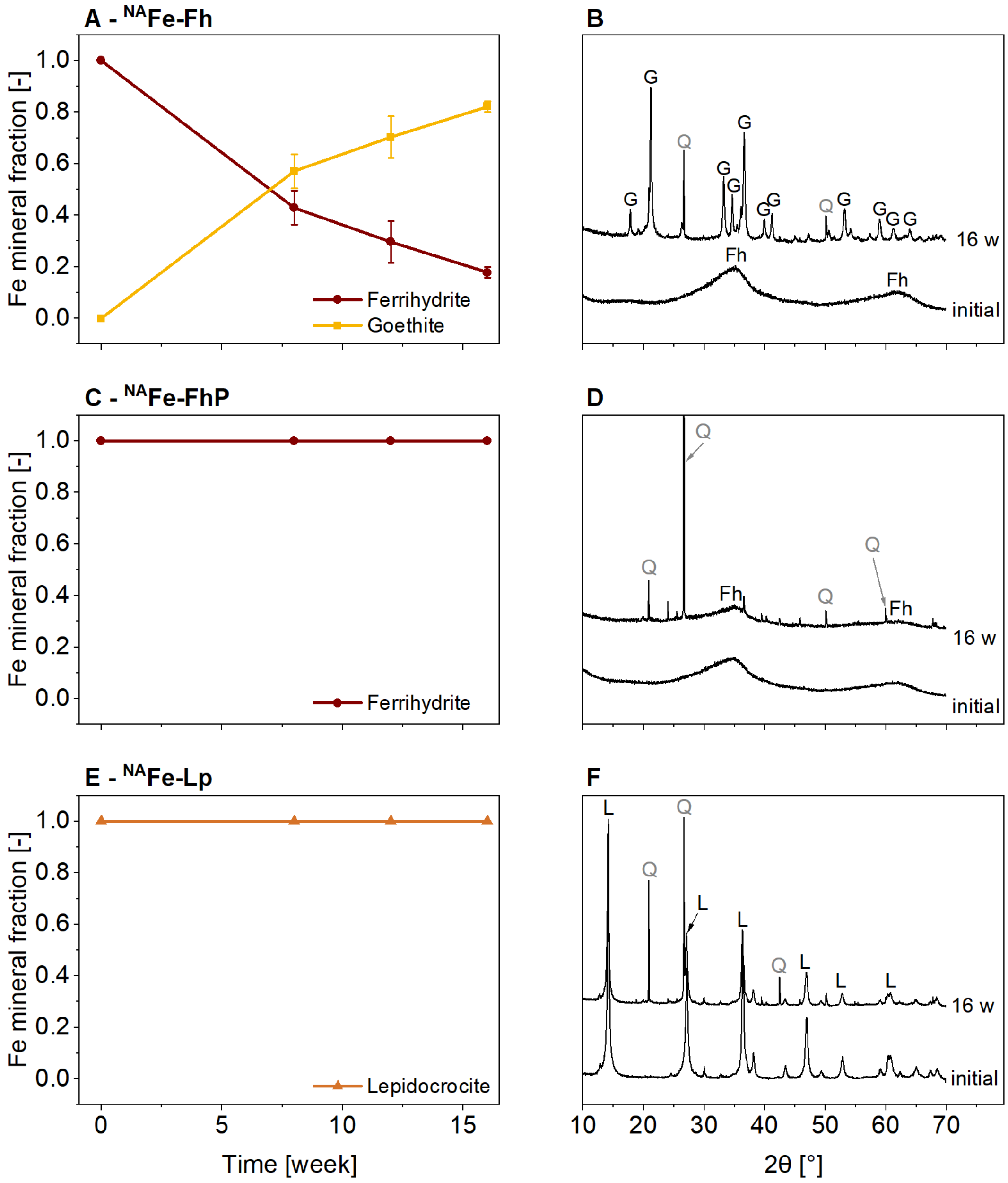

Figure S14: Mineral fractions in initial and field-incubated ^{NA}Fe ferrihydrite (A), ferrihydrite-P (C) and lepidocrocite (E) with corresponding X-ray diffraction patterns of initial and 16 week-incubated (16 w) ^{NA}Fe-Fh (B), ^{NA}Fe-FhP (D) and ^{NA}Fe-Lp (F). Error bars show the standard error of experimental triplicates, errors <0.02 are smaller than symbols and are not shown. Main diffraction peaks are labeled with Fh = ferrihydrite, G = goethite, L = lepidocrocite, Q = quartz. The quartz peak in panel D has been cut in height to improve the visibility of smaller peaks in the diffractogram. Fit results and parameters are presented in Table S7.

Fitting parameters of Rietveld quantitative phase analysis

Table S6: Fitting parameters and results from Rietveld fits of X-ray diffraction patterns collected from ^{NA}Fe-ferrihydrite (^{NA}Fe-Fh), ^{NA}Fe-ferrihydrite-P (^{NA}Fe-FhP) and ^{NA}Fe-lepidocrocite (^{NA}Fe-Lp) samples without soil, reporting the mineral fractions with the standard error between experimental triplicates. Mineral fractions are reported relative to the sum of Fe mineral fractions in the respective samples (i.e. quartz fractions not included). The preferred orientation (PO) of lepidocrocite and goethite, and fit evaluation parameters (R_{exp}, R_{wp}, GOF) were similar for experimental triplicates and are presented as averaged values. Abbreviations: w = week, PO = preferred orientation, Lp = lepidocrocite, Gt = goethite, Fh = ferrihydrite, se = standard error, R_{exp} = expected R, R_{wp} = weighted point R, GOF = goodness of fit.

Mineral	Time [w]	PO Lp 010	PO Gt 100	PO Gt 110	Fh	Fh_se	Lp	Lp_se	Gt	Gt_se	R_{exp}	R_{wp}	GOF
**^{NA}Fe-Fh**	8	-	1.09	0.58	0.43	0.07	0.00	0.00	0.57	0.07	5.36	10.03	1.89
	12	-	1.06	0.56	0.30	0.08	0.00	0.00	0.70	0.08	5.08	26.87	5.78
	16	-	1.05	0.63	0.18	0.02	0.00	0.00	0.82	0.02	5.40	9.18	1.70
**^{NA}Fe-FhP**	8	-	-	-	1.00	0.00	0.00	-	0.00	-	4.88	10.48	2.19
	12	-	-	-	1.00	0.00	0.00	-	0.00	-	5.13	7.83	1.53
	16	-	-	-	1.00	0.00	0.00	-	0.00	-	5.10	11.47	2.25
**^{NA}Fe-Lp**	8	0.95	-	-	0.00	-	1.00	0.00	0.00	-	5.44	11.39	2.10
	12	0.96	-	-	0.00	-	1.00	0.00	0.00	-	5.31	14.93	2.88
	16	0.97	-	-	0.00	-	1.00	0.00	0.00	-	5.22	15.20	2.90

S8. Element contents in incubated ^{NA}Fe minerals

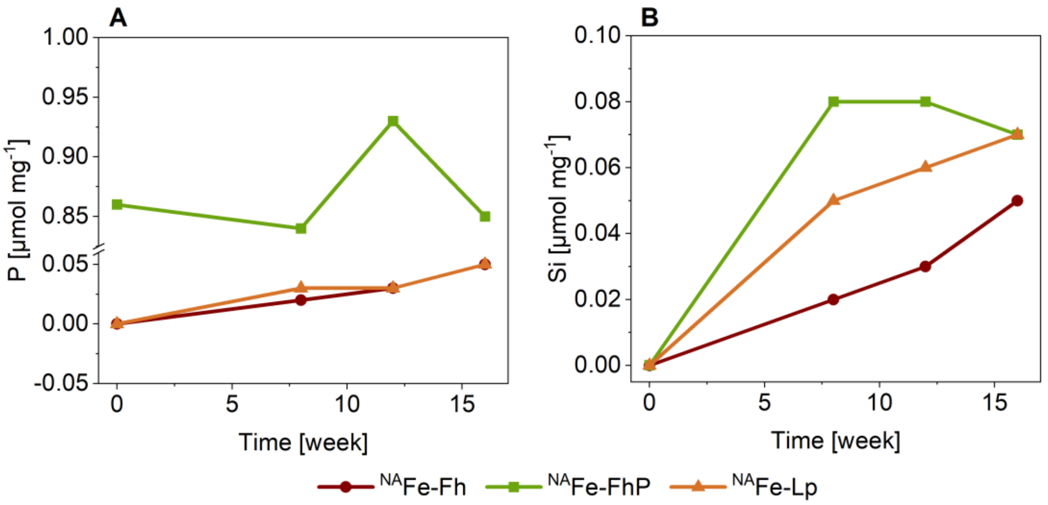

Figure S15: Element contents of phosphorus (P) and silicon (Si) in initial and incubated ^{NA}Fe ferrihydrite (^{NA}Fe-Fh), ferrihydrite-P (^{NA}Fe-FhP) and lepidocrocite (^{NA}Fe-Lp) samples without soil, determined after mineral dissolution in acid. The P/Fe ratio at 16 weeks was 0.01 for ^{NA}Fe-Fh and ^{NA}Fe-Lp, and 0.11 for ^{NA}Fe-FhP. The Si/Fe ratio at 16 weeks was 0.01 for ^{NA}Fe-Fh, ^{NA}Fe-Lp, and ^{NA}Fe-FhP.

S9. Aqua regia digestion results

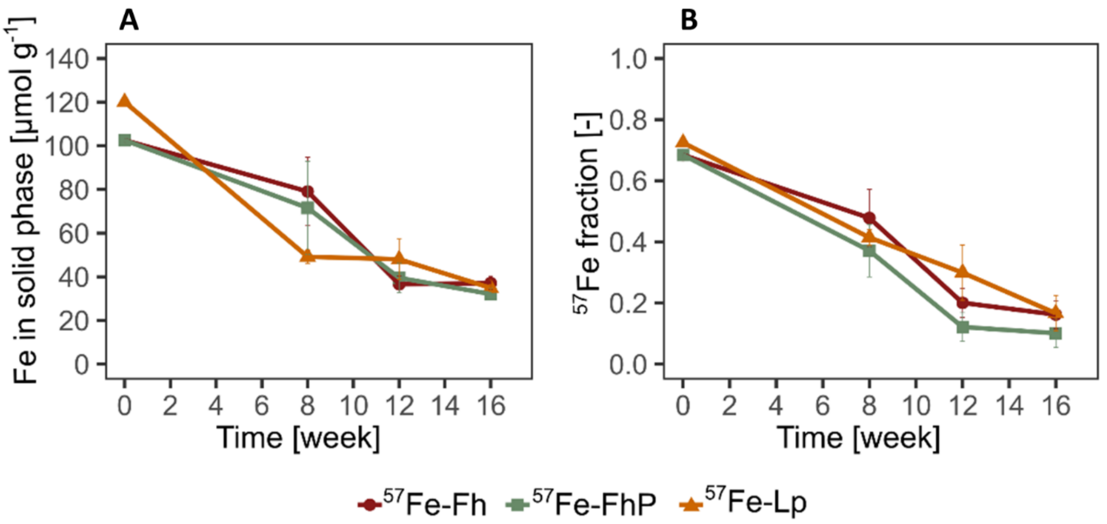

Figure S16: Fe concentration (A) and isotope fractions of ^{57}Fe (B) in ^{57}Fe-ferrihydrite-soil mixes (^{57}Fe-Fh), ^{57}Fe-ferrihydrite-P-soil mixes (^{57}Fe-FhP) and ^{57}Fe-lepidocrocite-soil mixes (^{57}Fe-Lp) after aqua regia digestion. Error bars indicate the standard error between experimental triplicates. The isotope fractions of ^{57}Fe (f^{57}Fe) in panel B are given relative to the sum of counts per second of ^{54}Fe, ^{56}Fe, ^{57}Fe, and ^{58}Fe.

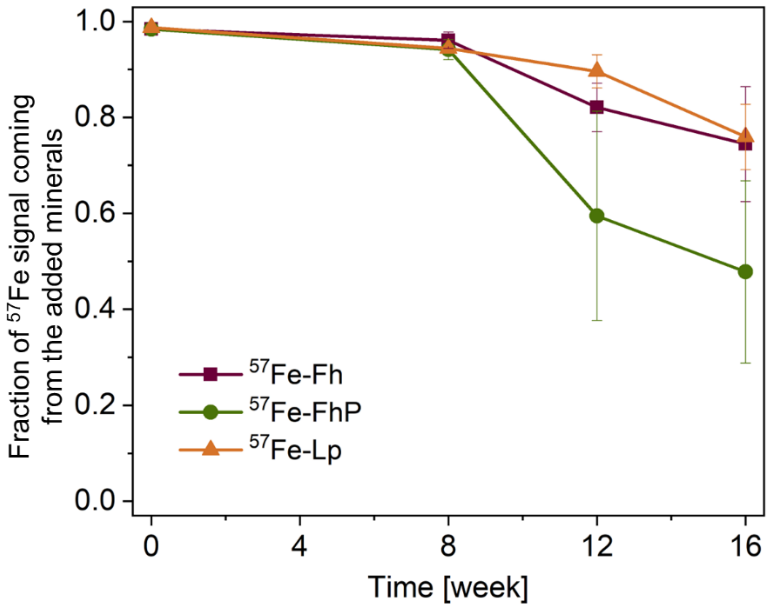

Figure S17: Fractions of ^{57}Fe atoms from added ^{57}Fe-labeled minerals in ^{57}Fe-mineral-soil mixes before and during the 16-week incubation in the field. The displayed fractions were calculated from Fe contents and ^{57}Fe fractions measured after aqua regia digestion of the mineral-soil mixes (Figure S16): Signal fraction_$^{57}Fe_{added\text{-}mineral}$ = 1-($^{57}Fe_{soil}$/$^{57}Fe_{mineral\text{-}soil\text{-}mix}$), where $^{57}Fe_{soil}$ is the content of ^{57}Fe in the soil in µmol g^{-1}, and $^{57}Fe_{mineral\text{-}soil\text{-}mix}$ is the content of ^{57}Fe in the mineral-soil-mix samples soil in µmol g^{-1}, as calculated from the Fe content and ^{57}Fe fraction after aqua regia digestion of the samples. The difference between fractions shown for ^{57}Fe-Fh and ^{57}Fe-FhP at 16 weeks is significant according to the Wilcoxon signed-rank test ($p = 0.03$) performed in RStudio 4.1.2.

Table S7: Aqueous iron (Fe) concentrations in initial and incubated ^{57}Fe-ferrihydrite (^{57}Fe-Fh), ^{57}Fe-ferrihydriteP (^{57}Fe-FhP) and ^{57}lepidocrocite (^{57}Fe-Lp)-soil mixes after the digestion with aqua regia, isotope fractions of ^{54}Fe (f^{54}Fe), ^{56}Fe (f^{56}Fe), ^{57}Fe (f^{57}Fe) and ^{58}Fe (f^{58}Fe), and the corresponding fraction of ^{57}Fe atoms in the sample coming from the initially added ^{57}Fe-labeled minerals (Frac. ^{57}Fe spike). Errors are given as standard errors (se) between experimental triplicates.

**Time**	**Fe**	***se***	***f*^{54}Fe**	*se*	***f*^{56}Fe**	*se*	***f*^{57}Fe**	*se*	***f*^{58}Fe**	*se*	*Frac. ^{57}Fe spike*	*se*
weeks	*µmol g^{-1}*		-								-	
**^{57}Fe-Fh**												
0	102.6	-	0.017	-	0.284	-	0.684	-	0.015	-	0.98	-
8	79.1	15.7	0.029	0.005	0.482	0.09	0.478	0.094	0.012	0.002	0.96	0.02
12	36.6	3.7	0.045	0.003	0.748	0.046	0.201	0.048	0.007	0.001	0.82	0.05
16	37.1	3.0	0.054	0.010	0.777	0.036	0.162	0.045	0.006	0.001	0.74	0.12
**^{57}Fe-FhP**												
0	100.6	-	0.016	-	0.279	-	0.690	-	0.015	-	0.98	-
8	71.6	21.3	0.035	0.005	0.585	0.082	0.371	0.085	0.010	0.001	0.94	0.02
12	39.5		0.050	0.003	0.824	0.045	0.122	0.047	0.005	0.001	0.60	0.22
16	32.1	2.8	0.051	0.003	0.843	0.045	0.101	0.047	0.005	0.001	0.48	0.19
**^{57}Fe-Lp**												
0	120.1	-	0.015	-	0.245	-	0.724	-	0.016	-	0.99	-
8	49.2	3.0	0.033	0.002	0.543	0.025	0.414	0.026	0.010	0.000	0.94	0.01
12	48.0	9.25	0.040	0.005	0.653	0.087	0.299	0.090	0.008	0.002	0.90	0.03
16	34.9	1.5	0.047	0.003	0.780	0.056	0.167	0.058	0.006	0.001	0.76	0.07

References

(1) Food and Agriculture Organization of the United Nations. *World Reference Base for Soil Resources 2014: International Soil Classification Systems for Naming Soils and Creating Legends for Soil Maps (Update 2015)*; 2014.

(2) Schulz, K.; Notini, L.; Grigg, A. R. C.; Kubeneck, L. J.; Wisawapipat, W.; ThomasArrigo, L. K.; Kretzschmar, R. Contact with Soil Impacts Ferrihydrite and Lepidocrocite Transformations during Redox Cycling in a Paddy Soil. *Environ. Sci. Process. Impacts* **2023**. https://doi.org/10.1039/d3em0314k.

(3) Rancourt, D. G.; Ping, J. Y. Voigt-Based Methods for Arbitrary-Shape Static Hyperfine Parameter Distributions in Mössbauer Spectroscopy. *Nucl. Instrum. Methods Phys. Res. B*, **1991**, *58* (1), 85–97. https://doi.org/10.1016/0168-583X(91)95681-3.

(4) Zhukhlistov, A. P. Crystal Structure of Lepidocrocite FeO(OH) from the Electron-Diffractometry Data. *Crystallogr. Rep.* **2001**, *46* (5), 730–733. https://doi.org/10.1134/1.1405857.

(5) Zepeda-Alarcon, E.; Nakotte, H.; Gualtieri, A. F.; King, G.; Page, K.; Vogel, S. C.; Wang, H.-W.; Wenk, H.-R. Magnetic and Nuclear Structure of Goethite (γ-FeOOH): A Neutron Diffraction Study. *J. Appl. Crystallogr.* **2014**, *47* (6), 1983–1991. https://doi.org/10.1107/S1600576714022651.

(6) Scarlett, N. V. Y.; Madsen, I. C. Quantification of Phases with Partial or No Known Crystal Structures. *Powder Diffr.* **2006**, *21* (4), 278–284. https://doi.org/10.1154/1.2362855.

(7) Aeppli, M.; Kaegi, R.; Kretzschmar, R.; Voegelin, A.; Hofstetter, T. B.; Sander, M. Electrochemical Analysis of Changes in Iron Oxide Reducibility during Abiotic Ferrihydrite Transformation into Goethite and Magnetite. *Environ. Sci. Technol.* **2019**, *53* (7), 3568–3578. https://doi.org/10.1021/acs.est.8b07190.

(8) Schulz, K.; ThomasArrigo, L. K.; Kaegi, R.; Kretzschmar, R. Stabilization of Ferrihydrite and Lepidocrocite by Silicate during Fe(II)-Catalyzed Mineral Transformation: Impact on Particle Morphology and Silicate Distribution. *Environ. Sci. Technol.* **2022**, *56* (9), 5929–5938. https://doi.org/10.1021/acs.est.1c08789.

(9) ThomasArrigo, L. K.; Byrne, J. M.; Kappler, A.; Kretzschmar, R. Impact of Organic Matter on Iron(II)-Catalyzed Mineral Transformations in Ferrihydrite–Organic Matter Coprecipitates. *Environ. Sci. Technol.* **2018**, *52* (21), 12316–12326. https://doi.org/10.1021/acs.est.8b03206.
